# What Nursing-Sensitive Outcomes Have Been Investigated to Date among Patients with Solid and Hematological Malignancies? A Scoping Review

**DOI:** 10.3390/nursrep13030096

**Published:** 2023-08-15

**Authors:** Chiara Visintini, Alvisa Palese

**Affiliations:** 1 Division of Hematology and Stem Cell Transplantation, Clinical University Hospital of Udine, 33100 Udine, Italy; chiara.visintini@asufc.sanita.fvg.it; 2 Department of Medical Sciences, University of Udine, 33100 Udine, Italy

**Keywords:** cancer care, onco-haematological care, oncology nursing, oncology nursing-sensitive outcomes, nursing-sensitive outcomes, outcome assessment, outcome measurement, satisfaction with care received

## Abstract

Nursing-sensitive outcomes are those outcomes attributable to nursing care. To date three main reviews have summarized the evidence available regarding the nursing outcomes in onco-haematological care. Updating the existing reviews was the main intent of this study; specifically, the aim was to map the state of the art of the science in the field of oncology nursing-sensitive outcomes and to summarise outcomes and metrics documented as being influenced by nursing care. A scoping review was conducted in 2021. The MEDLINE, Cumulative Index to Nursing and Allied Health, Web of Science, and Scopus databases were examined. Qualitative and quantitative primary and secondary studies concerning patients with solid/haematological malignancies, cared for in any setting, published in English, and from any time were all included. Both inductive and deductive approaches were used to analyse the data extracted from the studies. Sixty studies have been included, mostly primary (*n* = 57, 95.0%) with a quasi- or experimental approach (*n* = 26, 55.3%), conducted among Europe (*n* = 27, 45.0%), in hospitals and clinical wards (*n* = 29, 48.3%), and including from 8 to 4615 patients. In the inductive analysis, there emerged 151 outcomes grouped into 38 categories, with the top category being ‘Satisfaction and perception of nursing care received’ (*n* = 32, 21.2%). Outcome measurement systems included mainly self-report questionnaires (*n* = 89, 66.9%). In the deductive analysis, according to the Oncology Nursing Society 2004 classification, the ‘Symptom control and management’ domain was the most investigated (*n* = 44, 29.1%); however, the majority (*n* = 50, 33.1%) of nursing-sensitive outcomes that emerged were not includible in the available framework. Continuing to map nursing outcomes may be useful for clinicians, managers, educators, and researchers in establishing the endpoints of their practice. The ample number of instruments and metrics that emerged suggests the need for more development of homogeneous assessment systems allowing comparison across health issues, settings, and countries.

## 1. Introduction

Nurses make an essential contribution to healthcare quality, affecting patient, staff, and organisational outcomes [1]. Outcomes attributable to the care of nurses are described as nursing-sensitive outcomes (NSOs), a term first introduced by Maas et al. [2] and then defined as the behaviour, measurable condition, or perception of the patient or his/her family that is obtained or is significantly affected by the nursing care received [3].

In 1994, the American Nurses Association (ANA) launched the Nursing Safety and Quality Initiative to examine the impact of nursing structural and process variables in acute healthcare settings [4]. Since then, as NSO knowledge has been gradually established in the literature, different outcomes classification systems have been developed [5,6,7,8] by also investigating the contribution of some variables (e.g., skill-mix, working climate, leadership) [9,10,11]. In the emerging scientific debate, one of the biggest issues in outcomes research and evaluation that has been established is identifying relevant indicators, given their unstandardised definitions and lack of systematic collection by healthcare organisations. Nursing care is challenging to measure, and outcomes are often the result of a multidisciplinary team rather than of a single profession [1]. To map the state of the art on NSO research and to overcome its main intrinsic challenges, several reviews have been conducted in general nursing care [12,13] and in specific settings such as critical/intensive settings [14,15]. However, to our best knowledge, no reviews have been performed to date on oncology NSOs by including both hospital and outpatient settings.

The first attempt to measure NSOs in the oncological field was made by Oleske and Hauck (1988) [16], during which time interest in nursing outcomes was maturing [17]. The first narrative review was developed by Williams (1998) [18] to examine the dimensions of nursing care that contribute to patient perceptions of quality and caring behaviours. Later, between 2001 and 2002, the Oncology Nursing Society (ONS) Advanced Practice Nurse Retreat Project Team developed a work on nursing outcomes, generating the first list of NSOs in the field. These NSOs were categorised across patients, family, healthcare workers, symptoms, and financial aspects in cancer care, providing a description and an evaluation of the methodological and conceptual aspects of the definition for each of them [17].

Then, in 2003, the ONS Project Team defined NSOs as the results directly attributable to nursing care and the provision of nursing services or achieved in collaboration with other healthcare providers. The result of this working team was a classification scheme for outcomes influenced by oncology nurses; moreover, the ONS Project Team commissioned nursing research experts to develop evidence-based summaries on specific nursing-sensitive outcomes and discussed conceptual and methodological issues involved in measuring and affecting each of them [17]. In this context, Given and colleagues (2004) [19] provided five domains of NSOs containing patient- and provider-focused indicators, as follows:

1. symptom experience: pain, fatigue, insomnia, nausea, constipation, anorexia, breathlessness, diarrhoea, altered skin/mucous membranes, neutropenia;
2. functional status: ADL (activities of daily living), IADL (instrumental activities of daily living), role functioning, activity tolerance, ability to carry out usual activities, nutritional status;
3. safety (infections, falls, skin ulcers, extravasation incidents, hypersensitive reactions);
4. psychological distress (anxiety, depression, spiritual distress); and
5. economic (length of stay, unexpected readmissions, emergency visits, out-of-pocket costs, cost per patient day, and cost per episode of care).

Then, Griffiths et al. [20], funded by the National Cancer Action Team, developed a set of indicators in the field of outpatient oncology. These indicators were aimed at expressing the quality of nursing care capable of promoting improved patient outcomes. The authors summarised the indicators’ requirements and grouped them into the three broad areas of outcomes, safety, and structures–workforce [20]. Three years later, Griffiths and colleagues (2012) [21] produced a seminal scoping review to identify patient outcomes sensitive to the quality of nursing services in ambulatory cancer chemotherapy centres by including 28 studies published from 2002 to 2011. Patient experience, nausea, vomiting, mucositis, and safe medication administration emerged as the areas most sensitive to nursing care quality; however, no evidence associating the characteristics of the nursing services with outcomes were discovered [21]. More recently, Molassiotis et al. [22] performed a scoping review of 17 randomised clinical trials (RCTs)/cluster trials from 2001 to 2019 to explore the effectiveness of oncology nurse-led advanced practice for patients in cancer clinics and advanced practice in outpatient settings.

Alongside attempts to systematically develop a framework, several primary studies have considered NSOs in cancer care over the years—predominantly in acute care settings—by assessing the effectiveness of specific interventions to reduce pain [23,24], psychological distress [25,26], and fatigue [27], or to improve health-related quality of life (HRQoL) [28,29], the management of therapy-related side effects [30,31], the satisfaction with care received [32,33], and use of the healthcare system [16,34]. Most of these were based on the administration of validated self-report questionnaires, giving the patient the responsibility to assess the impact of nursing care received by embodying the patient-reported outcome measures (PROMs) approach. As shown in a recent systematic review [35], PROMs regarding HRQoL, general symptoms, and psychological distress are the most assessed in cancer care, and their reporting leads to a higher patient satisfaction, besides the improvement of patient symptom control. 

Updating the summaries available on the extent and nature of NSOs and their metrics, as documented to date in the whole pathway of oncology care, from hospital to outpatient clinics and care homes, may inform: (a) clinicians, regarding the outcomes that should be embodied in the minimum data set of care; (b) nurse managers, concerning the data that should be collected to monitor the quality of care [36]; (c) nurse educators, while planning undergraduate and advanced education programs; and (d) researchers, in defining evidence gaps [37] and which outcomes to scrutinise in their investigations. Thus, mapping the NSOs and their metrics as investigated to date regarding nursing care towards patients with solid and haematological malignancies (hereinafter, oncological care), in all settings, was the main intent of this study.

## 2. Materials and Methods

### 2.1. Research Questions

Three research questions were addressed: (1) How have NSOs been investigated in the field of oncological nursing care? (2) What NSOs have been measured to date in this research field and how? (3) To what extent does the NSOs framework in this field [19] reflect the nursing outcomes documented in the current literature?

### 2.2. Study Design

A scoping review was conducted in 2021, according to the framework proposed by Arksey and O’Malley (2005) [38] and recently revised by the Joanna Briggs Institute (JBI) [39]. There were specific steps followed: (a) identifying the research question according to the patient (P), concept (C), and context (C) framework; (b) identifying relevant studies; (c) selecting studies for inclusion; (d) collecting data; and (e) collating, summarising, and reporting the results. Methods and findings have been reported according to the revised *Preferred Reporting Items for Systematic Reviews and Meta-analysis Extension for Scoping Review* (PRISMA-ScR) statement [40] (see Tables S1 and S2).

### 2.3. Patient, Concept, and Context Framework

According to the JBI framework [37], the following elements were defined:

- Participants: adult patients with solid tumours or haematological malignancies, at their first diagnosis of cancer or at an advanced stage, in active therapeutic onco-haematological treatments.
- Concept: NSOs identified as any measurable behaviour, condition, or perception of the patient or his/her family that is obtained or is significantly affected by the nursing care received [3].
- Context: onco-haematological care settings, as studies conducted in cancer units/hospitals or outpatient centres, at public or private clinics, including teaching hospitals or at home. Hospice and/or palliative care services were not considered as context given that patients are not subjected to active therapeutic treatments.

### 2.4. Search Strategy

Following the JBI methodology [37], a three-step process was carried out. First, an initial search in the MEDLINE database (via PubMed) was performed in December 2020 by the first author to identify subject headings and keywords, for the well-refined search strategy. Secondly, the electronic databases MEDLINE (via PubMed), the Cumulative Index to Nursing and Allied Health (CINAHL), the Web of Science, and Scopus were examined up to 10 January 2021. Thirdly, the reference list of identified reports and articles was investigated to search for additional sources.

The following terms were considered in the search strategy: (1) “Outcome Assessment, Health Care”[Mesh] OR “Patient Outcome Assessment”[Mesh] OR “Patient Reported Outcome Measures”[Mesh] OR “Treatment Outcome”[Mesh] OR “Quality of Health Care”[Mesh] OR “Outcome Measures” OR “oncology outcome*” OR “Nursing sensitive outcome” OR “Nurse sensitive indicator”; (2) “Oncology Nursing”[Mesh] OR “oncological care” OR ((“haematological” OR” haematological”) AND “care”) OR “Nursing”[Mesh] OR “Nursing Care”[Mesh]; (3) “Oncology Service, Hospital”[Mesh] OR “Cancer Care Facilities”[Mesh] OR “cancer patient” OR ((“haematological” OR “haematological”) AND “patient”). All these terms and free-text words were combined into the search string with the Boolean operator ‘AND’. Duplicate articles were removed, and the reference list of secondary studies included was screened, with the purpose of identifying additional and relevant articles not recognised from the electronic databases.

### 2.5. Study Selection

All qualitative and quantitative primary and secondary studies, including research protocols, concerning adult patients (18+ years) with solid tumours or haematological malignancies, hospitalised or cared for at home, or as outpatients (e.g., ambulatory, day hospital) in public or private clinics, including teaching hospitals, and published in English at any time were eligible. Therefore, studies concerning: (a) paediatric, (b) palliative cancer as conditions where active therapeutic treatments are suspended, (c) oncological patients admitted to intensive care units, or (d) aspects different from nursing outcomes (e.g., diagnosis, therapy); and studies published (e) in languages other than English or (f) as abstracts, letters to the editor, book chapters, guidelines, comments, and editorials, were all excluded.

The first author (CV) screened article titles and abstracts according to the inclusion criteria; then, the second author (AP) independently screened the articles, reaching a consensus on eligible studies. All eligible articles were accessed. A full-text assessment was then performed by the first author (CV) independently, and then in common agreement with the second author (AP). The list of references of secondary studies was screened to retrieve relevant studies to add. Given the intent of the review, the quality of the included studies was not assessed [41].

### 2.6. Data Collection

A grid was developed in Microsoft Word^®^ and then pilot tested with 10 studies to ascertain its capacity to extract relevant data from the included studies. No changes were required, and the grid was then applied to all studies included. From each study the following data were extracted: (a) main study characteristics such as author(s), publication year, country, study aims and design, duration, sampling, setting/units involved, and participants; moreover, a brief description of the nursing care intervention delivered, when appropriate, was also extracted; (b) the NSOs as those outcomes reported in terms of definition and metrics used, data collection method/source and timing, and main results of the study. Data extraction was conducted by the first author (CV) and supervised in the entire process by the second author (AP).

### 2.7. Data Synthesis and Reporting

To answer the first research question, a synthesis of the main characteristics of the included studies was performed. Countries were categorised into seven geographical areas, according to the affiliation of the first author: Asia, Australia, Canada, Europe, Middle East, South America, and United States of America (USA). Then, studies were classified according to the pyramid of evidence [42] in secondary (meta-analyses, systematic reviews, and literature reviews) and primary studies, including interventional (RCTs, crossover, quasi-experimental, before-and-after studies), observational (retrospective and prospective cohort, cross-sectional, case-control), mixed-methods, and qualitative studies. The main study aims were summarised, whereas participants were categorised according to the site of the primary tumour (solid cancers or haematological malignancies) and the main patient profile (e.g., age, gender). Since none of the studies reported NSOs in relation to the different primary tumours (except for the descriptive cohort study by Gordils-Perez et al. [43]), we decided to present our results without categorising them according to the primary cancer. The study setting was categorised in hospital or clinical units/wards, outpatient (including ambulatory or day hospitals), or home care settings. The duration of the study was also summarised in terms of minimum and maximum time, as documented in each study.

To answer the second research question, an inductive approach was used. All NSOs were identified and then grouped according to their similarities and differences in sub-categories (e.g., ‘pain resolution’ and ‘satisfaction with pain management’ were grouped in the outcome ‘pain’). In each category, to map the main features of the NSOs as investigated to date, each outcome, as well as its frequency and metrics across studies (e.g., instruments, languages of tools), was reported. In the ‘Satisfaction with care’ category, considering NSOs as the extent of an individual’s experience with healthcare compared to his/her expectations [44], we also included the patient experiences that emerged from qualitative studies [45].

To answer the third research question, a deductive analysis approach [46] was applied, using the ONS framework [19], to compare the NSO sub-categories that emerged with the well-established framework in oncological care. The authors decided to consider the five domains (‘Symptom control and management’, ‘Psychological health status’, ‘Functional status’, ‘Safety’ and ‘Economic’ domains) taken from the ONS framework [19]. For those NSOs not fitting into one of the five ONS domains (e.g., ‘Satisfaction with care received’, ‘Quality of relationship with nurses’), the authors decided to include them under the domain ‘Other’.

In both approaches, the nurse-focused outcomes, defined as the improvement in nursing knowledge and skills, the enhancement of nursing participation in continuing professional development, and the increasing of nursing job satisfaction [47]—such as ‘Nurses’ understanding of the teach-back method’ [48], ‘Nurses’ knowledge’ and ‘Nurses’ satisfaction’ [30] were not included as they related to nurses as professionals. Furthermore, outcomes were also categorised as mono- or multidisciplinary; in the latter when they referred to other healthcare professionals (nurse assistants, physicians).

The first author (CV) summarised and synthesised the findings independently, before then agreeing with the second researcher (AP). Disagreements were discussed between the two (CV, AP). In case of potential disagreements, a third researcher was established to be involved upon request: however, no disagreements emerged between the authors.

## 3. Results

### 3.1. How Have NSOs Been Investigated in the Field of Oncological Nursing Care?

**Figure 1:**
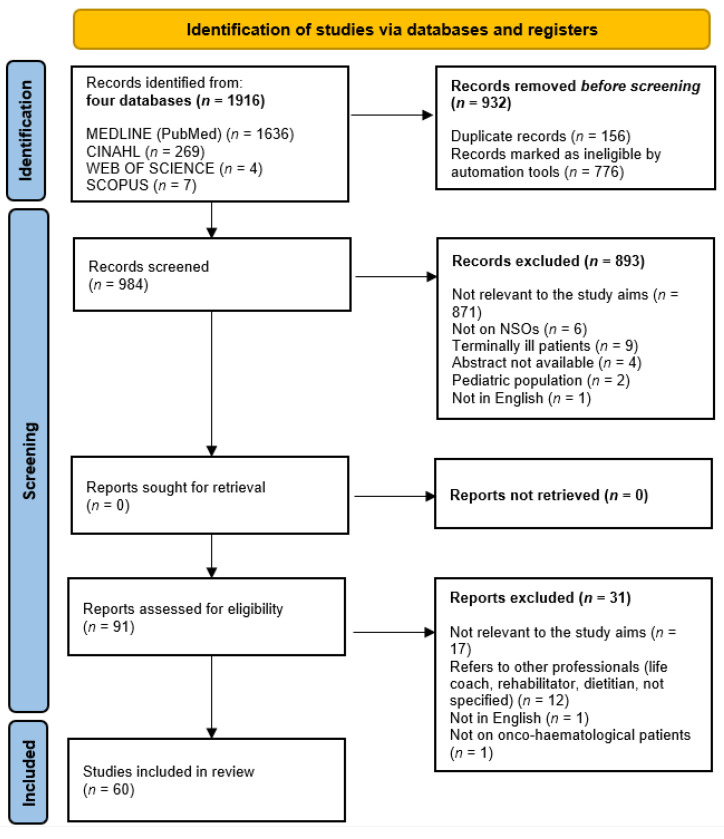
PRISMA-ScR 2020 flow diagram of the review process [40]. CINAHL: Cumulative Index to Nursing and Allied Health Literature; *n*: number; NSOs: Nursing-sensitive outcomes.

As reported in Figure 1, the 60 included studies were published between 1988 [16] and 2021 [49], mainly authored in Europe (*n* = 27, 45.0%), followed by the USA (*n* = 19, 31.7%), Asia (*n* = 4, 6.7%), Australia (*n* = 4, 6.7%), Canada (*n* = 3, 5%), the Middle East (*n* = 2, 3.3%), and South America (*n* = 1, 1.7%) (see Table S3).

Most of the studies (*n* = 47, 78.3%) were quantitative in nature (e.g., Given et al. [50]), while six (10.0%) were qualitative (e.g., Kvåle and Synnes, 2013 [51]), four (6.7%) were mixed-method studies (e.g., Bellomo, 2016 [52]), and three (5.0%) were secondary studies (one narrative review [18] and two scoping reviews [21,22]). Among the remaining, two (4.0%) were study protocols [28,52] and two were regarded as quality improvement projects [23,49].

Among the quantitative designs, experimental and quasi-experimental studies (*n* = 26, 55.3%), as RCTs (*n* = 11, 22.0%) (e.g., Du Pen et al. [53]), pre/post-test studies (*n* = 5, 8.3%) (e.g., Curcio et al. [54]), and quasi-experimental (*n* = 4, 6.7%) (e.g., Jakobsson and Holmberg, 2012 [55]), were reported. There were 21 (44.7%) observational studies, including cross-sectional and descriptive (*n* = 6, 12.0%) (e.g., Charalambous, 2013 [56]), prospective (*n* = 5, 10.0%) (e.g., Booth et al. [25]), correlational (*n* = 5, 10.0%) (e.g., Larsson et al. [57]), retrospective (*n* = 3, 6.0%) (e.g., Gray et al. [33]), and pro- and retrospective studies (*n* = 1, 2.0%) (MacLeod et al. [58]). Among the qualitative studies (*n* = 6, 10.0%), two were based on a phenomenological approach [51,59], one on grounded theory [60], and the remaining three were content [45,61] and thematic analysis based [62] (see Table S3).

Studies included from eight patients (exploratory descriptive [63]) to 4615 patients (cross-sectional study [64]). Considering a total population of 16,658 participants from 57 studies (data not available from Coolbrandt et al. [65], and the reviews by Griffiths et al. [21] and Molassiotis et al. [22]), the average age was 60.3 years (calculated from a population of 11,713, from 36 studies containing this data, missing in 24 studies). Studies included mainly females (*n* = 8879, 60.9%), as emerged from 46 studies including 14,568 individuals (data not available from 14 studies).

Patients were mostly affected by oncological malignancies (*n* = 24, 40.0%) (e.g., Hargie et al. [61]), followed by those affected by both cancer and haematological issues (*n* = 12, 20.0%) (e.g., Jakobsson and Holmberg, 2012 [55]) and by haematological issues alone (*n* = 9; 15.0%) (e.g., Braamse et al. [28]); however, data regarding the primary tumour site were not available from a quarter of the studies (*n* = 15, 25.0%) (see Table S3).

Most of the studies were conducted in hospital or clinical units/wards (*n* = 29, 48.3%) (e.g., Charalambous, 2013 [56]), followed by outpatient settings, such as ambulatory or day hospitals (*n* = 21, 35.0%) (e.g., Given et al. [50]) and homecare settings (*n* = 4, 6.7%) (e.g., De Veer et al. [64]). In four studies (6.7%), data were collected from a combination of these different settings (e.g., Blackburn et al. [23]); however, data were not available from two studies (3.3%).

The study durations ranged from 2 weeks in the exploratory descriptive study by Krishnasamy (1996) [63] to 43 months in the study protocol designed by Braamse et al. [28], although 14 studies (23.3%) did not report the study duration. The reviews by Griffiths et al. [21] and Molassiotis et al. [22] included studies from 2002 to 2011 and from 2001 to 2019, respectively.

At the overall level, 33 studies (55.0%) were aimed at evaluating the effectiveness of an intervention, such as structured nurse leader rounds [32], use of comfort kits [23], nursing telephone consultations [34], or consumption of boiled or congee potatoes [66], while the remaining (13, 21.7%) analysed the effectiveness of the introduction of new nursing roles, such as a clinical nurse specialist [25], a certified oncology nurse [30], and a nurse oncology navigator [43], or nurse-led clinics [58]. Then, twelve studies (20.0%) were aimed at evaluating the satisfaction of care received (e.g., Charalambous, 2013 [56]) and two studies (3.3%) at assessing screening tools [26,67].

### 3.2. What NSOs Have Been Measured to Date and How? The Inductive Approach

As reported in Table 1, the included studies (N = 60) documented a total of 151 NSOs, on average 2.3 per study. These NSOs have been grouped into 57 sub-categories and 38 categories.

The most investigated category outcome was ‘Satisfaction and perception of nursing care received’ (*n* = 32, 21.2%), with a range of instruments and different study designs (e.g., pre/post-test study design), including 10,253 patients, mainly with solid cancer, at the hospital level, in Europe (see Table S4). The ‘Nursing care process quality’ (*n* = 18, 11.9%) was the second most investigated outcome, also in this case with different study designs and instruments, including 3884 patients, mainly with solid cancer, across European hospitals. As the third most investigated outcomes, ‘Psychological distress’ and ‘Experiences with therapy-related side effects’ (*n* = 11, 7.3%) were investigated with a variety of tools, mainly in RCTs, covering over 2000 patients with solid/haematological tumours, also across European hospitals. Single outcomes (such as ‘Comfort’, ‘Coping’, ‘Constipation’, ‘Problem solving ability’) have been reported in 20 studies (33.3%), while 20 multidisciplinary outcomes emerged (13.2%) (physicians, nurses, psychologists, …) (see Table S4).

Regarding outcome metrics systems (*n* = 133), mainly self-report questionnaires (*n* = 89, 66.9%), e.g., [30,68], mostly validated (*n* = 56, 42.1%) (e.g., the Comprehensive Assessment of Satisfaction with Care [69]), were used; however, administrative data (*n* = 15, 11.2%), e.g., the number of emergency department visits or hospital admissions [34]), were also used, in addition to clinical objective measures (*n* = 8, 6.0%) (e.g., complication rates [16]). Patient narratives (*n* = 7, 5.2%) regarding experiences, perceptions, and reflections on nursing care received emerged from semi-structured interviews and comments, e.g., [60] (see Table 1 and Table S4).

### 3.3. To What Extent Does the NSOs Framework in This Field Reflect the Nursing Outcomes Documented to Date? The Deductive Approach

According to the ONS outcome classification [19], the most investigated outcomes regard the ‘Symptom control and management’ domain (*n* = 44, 29.1%), followed by the ‘Functional status’ and the ‘Economic’ domains (both *n* = 20, 13.2%), the ‘Psychologic health status’ (*n* = 13, 8.6%), and the ‘Safety’ domains (*n* = 4, 2.6%). The remaining 50 (33.1%) NSOs were not categorisable in the given domains; thus, these have been considered as ‘Other’ (see Table 2).

The NSO most investigated, belonging to the ‘Symptom control and management’ domain, was ‘Pain’ (*n* = 10, 6.6%), mainly in its resolution or reduction due to nursing interventions (*n* = 7, 4.6%), followed by ‘Fatigue’ (*n* = 3, 2.0%) and ‘Nausea and vomiting’ (*n* = 2, 1.3%). However, more general therapy-related symptoms, not directly included in the ONS provided exemplars [19] were reported eight times (5.3%). On the other hand, in the ‘Economic’ domain, ‘Timing of the care process’ and ‘Emergency department visits’ were the predominant NSOs (both *n* = 4, 2.6%).

Withing the ‘Functional status’ domain, ‘Role functioning’ was the most documented according to the ONS exemplars (*n* = 3, 2.0%), while HRQoL, reported eight (5.3%) times, was also mentioned. Studies reporting outcomes referring to the ‘Psychological health status’ domain focused mainly on psychological distress, in general, affecting oncological patients (*n* = 7, 4.6%) with respect to ‘Anxiety’ and ‘Depression’ alone (*n* = 2, 1.3%, each).

Studies that documented outcomes negatively influencing the ‘Safety’ domain (*n* = 4, 2.6%) included central venous catheter-related infections (CRIs), falls, safe medication administration, and unplanned therapy interruptions (0.6%, each). Finally, nearly one-third of the NSOs (*n* = 50, 33.1%), belong to the ‘Other’ domain, including ‘Satisfaction with care received’ (*n* = 19, 12.6%) and ‘Patients’ experiences and perceptions of care received’ (*n* = 11, 7.2%), followed by ‘Quality of care received’ (*n* = 5, 3.3%).

## 4. Discussion

### 4.1. Characteristics of Included Studies

An impetus on oncology NSOs in the last decade is visible, considering that Williams (1998) [18] included three studies, Griffiths et al. [21] 28 studies, and Molassiotis et al. [22] 17 studies. In our scoping review, there were 60 studies, mainly performed across Europe, which seems to have played a leader role in this field of research; on the other hand, this could limit the generalizability of the findings to other geographic locations given also both the cultural and educational influences on patients’ experiences regarding the nursing care received. However, no international primary study has emerged to date, suggesting an area of improvement, given that differences in policies, education, roles, and skill-mix [70] might influence the outcomes.

Most studies were based on quantitative designs, mainly with the purpose to evaluate the effectiveness of interventions. Since Griffiths and colleagues (2012) [21], around ten years ago, provocatively concluded their scoping review by indicating that an outcome sensitive to nursing care is not always an NSO, meaning that not all the evidence was considered as sensitive quality measurements for NSOs, more efforts have been undertaken recently in this research field.

Studies included largely women and adult patients, with a mean age of 60.3 years. Given that cancer has become a chronic condition with prolonged life expectancies [71], more older individuals are expected to be involved [72] in future studies. Moreover, studies about oncological patients alone represented almost three times the number of studies on hematologic neoplasms. This reflects the higher prevalence of solid compared to blood tumours [73]. Future studies on NSOs should consider the benefits of including patients with different diseases as comorbidities and the need to stratify the sub-groups when a mix of populations are involved and to highlight variations in NSOs across different health conditions. Moreover, hospitals and clinical units/wards were the prevalent settings in the available studies. A few studies investigated NSOs in the home care setting and in the continuum of care integrating hospital, outpatient, and home care-based contexts [60] suggesting also in this case an area of improvement. Finally, the quality of the reporting should be improved given the amount of missed data detected across the retrieved studies, e.g., Bellomo (2016) [52]. Developing a minimum data set framework to support NSOs investigations in this field of research and in other fields might be important.

### 4.2. What NSOs Have Been Measured to Date and How? The Inductive Approach

From our analysis, 151 NSOs emerged, more than two in each study on average, which have been inductively categorised into 38 categories and 57 subcategories. A similar exercise to discover the state-of-science in this field was conducted in the intensive care context [14,15], where among adults, from 1996 to 2019, 233 NSOs emerged and were grouped into 35 categories [14]. By scrutinizing Table 1, it is possible to further categorise the NSOs that emerged as objective (e.g., health care devices), subjective (e.g., psychological aspects of the patient or the nurse), and/or somatic (e.g., illness and the patient’s body). However, the emerged NSOs were left granular to render available the full list for different users such as researchers, clinicians, and managers while designing their studies, electronic records, and metrics to assess the effectiveness of quality improvement strategies. Above all, these findings suggest two lines of discussion: the first methodological and the second regarding contents.

Starting with the methodological discussion, oncological nursing care affects a variety of outcomes, suggesting a complexity of care that may affect different dimensions; therefore, studies should embody this complexity by identifying a range of outcomes capable of expressing the full contribution of the nursing care. At the overall level, there emerged categories that include several sub-categories (such as ‘Nursing care process quality’ or ‘Health care system utilisation’) and those not reporting any sub-category (‘Health-related quality of life’ or ‘Self-efficacy’). This seems to suggest that there are NSOs that are well-articulated in several specific aspects of the same domain (e.g., ‘Satisfaction with nursing care’) and others that are not. The progressive advancement of knowledge may nurture the latter with more declinations, providing sub-categories capable of detecting peculiar aspects. Continuously updating the reviews by inspecting these changes may contribute to advancements in the field of NSOs research. Furthermore, a rich variety of metrics have emerged across the categories, of which the majority were validated self-report instruments, despite nursing care having been underlined as difficult to measure [1]. The ample use of self-report questionnaires as primary methods of data collection in this field should be considered also for their research implications regarding the degree of subjectivity or potential bias [74]. However, mapping the tools may be useful for researchers in identifying the most suitable instruments for each NSO they intend to measure. Unfortunately, the variety that emerged may prevent any form of comparison or summary of the effects of specific interventions (e.g., with a meta-analysis), suggesting that specific tools should be recommended for each NSO according to their properties.

Data on some NSOs have been provided from large populations (e.g., ‘Satisfaction and perception of nursing care received’, covering more than 10,000 patients), whereas other outcomes only from a small population (‘Uncertainty’, 146 patients), or were not well described in their main demographic/health issues. First, some outcomes are classic or popular (e.g., ‘Satisfaction’) and mostly investigated as compared to others, suggesting the need to assess if they really reflect the specific nursing care contribution in this setting. Continuing to investigate some outcomes may affect the accumulation of knowledge in favour of some while limiting others; moreover, it may also affect the external validity of the studies performed in the content of NSOs, when the detailed description of the populations involved is unclear. A few outcomes have been investigated mainly at only one level (e.g., ‘Role functioning’ in hospital units), while the majority have been investigated in mixed settings (e.g., ‘Satisfaction and perception of nursing care received’, ‘Nursing care process quality’, ‘Experiences with therapy-related side effects’). Likewise, more clarity regarding the settings is recommended, given the lack of information reported in some studies.

Twenty (13.2%) multidisciplinary outcomes emerged, suggesting that some NSOs are ‘pure’ and others are ‘multidisciplinary’, as investigated strictly related to the nursing care provided and in combination with other professionals, respectively. However, some NSOs have been investigated in both manners (e.g., ‘Patients’ satisfaction with care’). Sometimes it is difficult to attribute an outcome to a single profession [1]. As stated by Given and Sherwood in their ‘White Paper’ (2005) [17], measures most useful for evaluating multidisciplinary interventions where nursing care plays more of a leadership role are suggested.

At the content level, the most reported outcome was ‘Satisfaction and perception of nursing care received’ in nearly half of the studies. Patient satisfaction has been recognised as a quality indicator sensitive to nursing care by the ANA since 1995 [4] and nowadays it is considered a PROM measure [75]. ‘Nursing care process quality’, as it is also called by several stakeholders (e.g., legislators, health agencies, regulatory authorities) was the second NSO evaluated in terms of frequency [17]. According to the findings, this NSO was described by a wide variety of indicators, such as self-validated questionnaires for caring behaviours, proxy measures such as the length of patient stay, the time from the first nurse visit to the last, or the number of contacts with nurses. The intrinsic nature of patient experience as both an aspect and an indicator of quality was also underlined by Griffiths et al. [21], and other examples to increase the nursing quality of care are offered in the literature [76].

‘Psychological distress’, as the third most investigated outcome, is in line with the uncertainties and fears experienced by a person diagnosed with cancer, modulated when they receive relational and supportive care [77]. Equally investigated was the NSO ‘Experiences with therapy-related side effects’, which could have been underestimated, possibly because cancer-related symptoms have been considered as a whole (e.g., ‘Symptom distress’ [78]) rather than a specific issue (e.g., ‘Constipation’ [66]). Finally, the next most investigated, ‘Pain’—assessed in 10 (16.7%) studies—should be considered a multidisciplinary outcome [21], as likely should ‘HRQoL’ [79]. The latter might be particularly important when disease-focused end points show minimal or modest differences and where outcomes need to be addressed by symptom relief or improving or maintaining function, thus impacting HRQoL instead of the disease progression [17].

### 4.3. To What Extent Does the NSOs Framework in This Nursing Field Reflect the Nursing Outcomes Documented to Date? The Deductive Approach

In accordance with the latest ONS classification [19], the most investigated outcomes are in the ‘Symptom control and management’ domain, in line with Griffiths et al. [21], where nausea, vomiting, and mucositis appeared as the outcomes most likely sensitive to nursing in ambulatory care, suggesting that nursing interventions play a vital role in preventing or minimising symptoms and complications [17]. These clinical outcomes are easily accessible through patient clinical records and hospital discharge registers [80,81]; in addition, they make the nursing involvement in patient care explicit, suggesting the essential role of nurses in improving symptom control and management related to cancer treatments. On the other hand, the NSOs that emerged in our review were mainly categorised under the ‘Other’ domain, with ‘Satisfaction with care received’ as the most investigated. Most of the included studies were published after 2004—the year of the framework by Given et al. [19]. This seems to suggest that the framework probably needs to be updated, reflecting the expansion of the evidence available and the expansion of the role of nurses that might be different in terms of responsibilities and actions as compared to that documented nearly 20 years ago (e.g., the management of sexual problems in cancer patients [82]).

The ‘Economic’ and the ‘Functional status’ domains were equally represented. Considering the chronic and debilitating nature of cancer, greater healthcare service utilisation, including emergency department visits and inpatient hospitalisations, is a significant contributor to the growing costs of cancer care [83,84]. Research should assess interventions capable of minimising the negative individual and health service-related costs; moreover, the need to investigate the impact of nursing care on functional status is also important, given that older cancer patients often have comorbidities, and they are more vulnerable to functional impairments during chemotherapy [85].

The ‘Psychological health status’ domain emerged with anxiety, depression, and psychological distress, in general, as the most frequent outcomes. Also ‘The Patient-Centered Outcomes Working Group Report’ by Bevans et al. [79]—assessing common patient-centred outcomes among haematological survivors within one year after transplantation—reported high rates of prevalence. However, this was not defined as an NSO. This domain should be more often considered in the future, with studies developed in cooperation with psychologists and other professionals, besides always considering psychological distress when caring for oncological patients. Finally, studies included in the ‘Safety’ domain emerged but with less frequency, suggesting the need to continue to investigate these NSOs to promote a robust culture of safety and to prevent complications.

### 4.4. Limitations

This scoping review has several limitations. Firstly, although based upon a systematic approach, some studies could have been missed; moreover, a selection bias related to some limitations (e.g., English language) may have led to some studies being missed. In addition, the lack of a clear definition of NSO has challenged the study identification, eligibility, and inclusion, further introducing risk of selection bias. Secondly, while establishing the inclusion and exclusion criteria, we excluded those studies regarding terminally ill patients or those receiving palliative care given that specific outcomes are pursued in this population (e.g., the problems related to the relational dimension, both with patients and their families). Future and specific reviews are called for in this field to complete the summary of data on NSOs also in this population.

Thirdly, the categorisation processes conducted might have been influenced by the researchers’ experience, background, and subjective interpretation, leading to potential biases in detecting and categorising outcomes. Furthermore, we decided to use the ONS framework [19], which, to the best of our knowledge, is the latest in the oncological field. Fourthly, due to the high number of the included studies, the heterogeneity of the research methods used and the nature of the scoping review itself, the methodological quality was not assessed [41]. In this context, we used the pyramid of evidence by Thiese (2014) [42] as a general reference point to organise the studies retrieved in a logic form; however, the knowledge extracted by each study was not weighted according to its position in the pyramid of evidence.

## 5. Conclusions

Research into NSOs in onco-haematological care is characterised mainly by quantitative study designs, conducted across European hospitals, predominantly including adult females. In the inductive analysis, 151 outcomes emerged that were grouped into 38 categories. Some NSOs were well explored (e.g., ‘Satisfaction and perception of nursing care received’, ‘Nursing care process quality’, ‘Psychological distress’, ‘Experiences with therapy-related side effects’, ‘Pain’, and ‘Health-related quality of life’), while others have been reported only in a few studies (e.g., ‘Comfort’, ‘Coping’, ‘Constipation’). On the other hand, in the deductive analysis categorising the NSOs that emerged into the ONS classification framework, the ‘Symptom control and management’ domain was the most documented; however, an additional category was needed to include the majority of NSOs that were not includible in the available framework (i.e., ‘Other’).

Thus, according to the findings, there is a need to further develop the ONS conceptual framework beyond the identification of new lines of outcome-related research, and to cover the gaps that have emerged. In this context, given the complexity of cancer care, whose outcomes are often the result of teamwork, more in-depth investigation of the nursing contribution made inside team care activities could be important. Moreover, an ample number of instruments have emerged for the same NSO category, mainly using patient-report questionnaires, suggesting a need for more homogeneous measurement and assessment systems, to allow comparison across settings, irrespective of country. 

The findings may be useful to inform oncology nurses regarding the data to be recorded in clinical documentation. Debating which NSOs to include in electronic administrative databases could support the systematic evaluation of outcomes at the unit and system levels, along with benchmarking. Oncological advanced practice nurses and researchers should lead the initiative of transferring NSOs into clinical practice, especially regarding those NSOs more investigated across the literature, such as ‘Satisfaction of nursing care received’. From an educational point of view, the set of NSOs could be considered as core content in the nursing curricula, at both under- and post-graduate levels, and more awareness of outcomes measurement and implications must be addressed in policies, trusts, and public health issues. Finally, regarding policy decisions, these results may be useful as data to be collected and included in data sets to record and document the nursing care provided and its impact, facilitating decisions for healthcare systems. Furthermore, the design and implementation of a nursing minimum data set in onco-haematological care is desirable at the international level. Lastly, emphasis on NSOs could also have implications for public opinion in recognising the role of oncology nurses in delivering high-quality care.

## Figures and Tables

**Table 1:** Summary of NSOs (N = 151) as investigated to date in the included studies (N = 60).

NSOs: Categories (*n*, %)	NSOs: Sub-Categories (*n*, %) *	Metrics of the NSOs: Data Collection Tool and Language * (*n* of Studies)
Satisfaction and perception of nursing care received (32, 21.2%)	Satisfaction (19, 12.6%) *	PSNCQQ, validated questionnaire, Arabic (1) GGZ-thermometer, validated questionnaire, Dutch (1) PSS, self-report validated questionnaire, Greek (1) PGI, validated questionnaire, English (2) Non-declared tool, English (1), NA (1) Ranking questionnaire, 5-point scale, not validated, English (1) Satisfaction survey, English (1) 5 A’s model’, self-report questionnaire + 11-point Likert-type scale, self-report questionnaire, Dutch (1) VAS tool, Japanese (1), NA (1) 7 questions from the PGO, not validated self-report questionnaire, English (1) PSCC, validated scale, English (1) Satisfaction questionnaire, previously piloted self-report questionnaire, Spanish (1) HCAHPS, self-report questionnaire, English (1) PSQ III, validated questionnaire, Dutch (1), English (1) PASQOC, validated self-report questionnaire, German (1) * NSC, validated self-report questionnaire, Swedish (1) CASC, validated self-report questionnaire, Swedish, and two open questions (1) APS POQ, validated tool, Greek (1) * Open questions and semi-structured interviews (1)
	Experiences and perceptions of care (11, 7.2%) *	Non-declared tool, English (1) 7 single questions adapted by the 10-care dimension by NCSR, not validated, Greek (1) Survey questionnaire, non-validated, English (1) * Non-validated self-report questionnaire with Likert scales and open-ended comments, English (1) * 6-items of study specific questionnaire, Swedish (1) Semi-structured interview analysed using a qualitative content analysis (1) * Semi-structured interviews analysed by thematic analysis (1) * NA (1)
	Quality of the relationship with nurses (2, 1.3%) *	Deep-probe semi-structured “sensemaking” interviews (1) Not highly structured interview using an hermeneutical approach (1) *
Nursing care process quality (18, 11.9%)	Quality of care received (5, 3.3%)	In-depth dialogues analysed by thematic analysis (1) CBI-24, validated self-report questionnaire, English (1) QPP, validated self-report questionnaire, Swedish (1) ICS, self-report validated questionnaire, Greek (1) QONCS, self-report validated questionnaire, Greek (1) SMAT, 8 item tool to analyse audio-recorded calls and documentation, English and French (1)
	Timing (5, 3.3%)	Time from diagnosis to treatment (1) Waiting time from admission to transfusion (1) Length of patient stay (1) Time conducting the screening in the ambulatory (1) Time from the first health nurse visit to the last home health nurse visit (1)
	Caring and uncaring behaviours (4, 2.6%) *	CARE-Q, validated assessment tool, Chinese (1) and Norwegian (1) Semi-structured interview analysed by thematic analysis (1) * HCI, validated questionnaire, English (1)
	Access to care (2, 1.3%)	Time for the first patient call to first oncology provider consultation (1) 7-items of study specific questionnaire for access to resources (1)
	Acceptability and accessibility of cancer service (1, 0.6%) *	Semi-structured interviews analysed by grounded theory (1) *
	Disposition at discharge (1, 0.6%)	Disposition at discharge (1)
Psychological distress (11, 7.3%)	General (7, 4.6%)	HADS, self-report validated questionnaire, English (2), Dutch (1), Swedish (2), NA (1) PHQ-9, self-report questionnaire, Dutch (1), English (1) * POMS, validated, NA (1) NCCN DT, self-report validated questionnaire, English (1) The Problem List, self-report validated questionnaire, English (1)
	Depression (2, 1.3%) *	CES-D, self-report questionnaire, English (1), NA (1)
	Anxiety (2, 1.3%)	STAI-state adapted, self-report validated questionnaire, Dutch (1), NA (1) GAD-7, validated self-report questionnaire, English (1)
Experiences with therapy-related side effects (11, 7.3%)	General symptoms (8, 5.3%) *	SES, 5-point scale, English, and telephone-based interview (1) MDASI, validated self-report questionnaire, English (1) MDASI-NH, self-report questionnaire, English (1) FACT-HNSI, validated, NA (1) CSAS, validated, NA (1) MSAS, validated questionnaire, English (1) * NA (1) NRS, validated, NA (1) Checklist format using based on scientific literature, Portuguese (1) Frequencies from the patients’ diaries (1)
	Symptom distress (3, 2.0%)	SDS, self-report questionnaire, English (3) *
Health care system utilization (10, 6.6%)	Emergency department visits (4, 2.6%)	N of emergency department visits (4)
	Hospital admissions/hospitalizations (2, 1.3%)	N of hospital admissions/hospitalizations (2)
	Referrals (1, 0.6%)	Referral rate (1)
	Clinical appointments/visits (3, 2.0%)	N of appointments (1) N of home visits (1) N of missed appointments (1)
Pain (9, 6.0%)	Resolution/reduction (7, 4.6%) *	BPI-C, self-report questionnaire, Chinese (1) and English (2) * VAS, self-report validated tool (1) * 10 degree not-declared scale, English (1) NA (1)
	Satisfaction with pain management (2, 1.3%)	Non-declared scale for ambulatory patients and two statements for inpatients (1) RPS-POQ, validated self-report questionnaire, Hebrew (1)
Health-related quality of life (8, 5.2%)	-	EORTC QLQ-C30, validated questionnaire, Dutch (1), NA (1) SF-8, validated questionnaire, Japanese (1) SF-36, validated questionnaire, Dutch (1), English (1) * EORTC QLQ-C15-PAL, validated questionnaire, Dutch (1) FACIT-Sp, validated self-report questionnaire, Swedish (1) FACT-G, validated questionnaire, English (2), NA (1) FACT-HN, validated, NA (1) FACT B/ES, validated, NA (1) QLQ-BR23, validated, NA (1)
Barriers and facilitators to intervention adherence/symptoms (5, 3.3%) *	-	ASK-12, validated tool, English (1) PBS, validated questionnaire, English (1) * BQ-SF, validated self-report questionnaire, Hebrew (1) Checklist and open-ended questions, English (1) * Self-report questionnaire developed for the study, English (1)
Role functioning (4, 2.6%)	Functional status (3, 2.0%) *	Two subscales from the SF-36, validated questionnaire, English, and telephone-based interview (1) ESDS, self-report questionnaire, validated, English (2) *
	Ability to function independently (1, 0.6%)	DGSS, validated questionnaire, Dutch (1)
Knowledge in (4, 2.6%)	Understanding diagnosis and disease management (2, 1.3%)	Developed survey, English (1) Knowledge questionnaire derived from Miller (2008), English (1)
	Managing chronic cancer pain (1, 0.6%)	PPQ, validated questionnaire, English (1)
	Medications and side effects (1, 0.6%)	Two items of study questionnaire, English (1)
Self-efficacy (4, 2.6%)	-	NA (1) PAM, validated self-report questionnaire, Dutch (1) SEMCD 6, validated questionnaire, English (1) Two subscales of the study questionnaire (1)
Fatigue (3, 2.0%)	-	FACT-F, validated scale, Spanish (1) BFI-I, validated, English (1), French (1)
Health status (3, 2.0%) *	-	SF-12, validated questionnaire, English (1) 28-item retrospective survey, not validated, English (1) * HADS, validated questionnaire, English (1) EORTC QLQ-C30, validated questionnaire, English (1)
Activation (2, 1.3%)	-	5-items of study specific questionnaire, Swedish (1) PAM, validated self-report questionnaire, Dutch (1)
Awareness of the importance of received intervention (2, 1.3%)	-	11-point Likert-type scale, self-report questionnaire, Dutch (1) Ranking questionnaire, non-validated, English (1)
Clinical effectiveness of intervention delivered (2, 1.3%)	Capecitabine management (1, 0.6%)	Capecitabine dosing schedule, N of treatment modifications, N of response to treatment, N of adverse events, N of need for consultations with general practitioners (1)
	Incidence of CRIs (1, 0.6%)	Clinical criteria and laboratory data of CRI (1)
Oral care (2, 1.3%)	Oral situation (1, 0.6%) *	OAG, non-validated assessment tool, Swedish (1) *
	Mucositis (1, 0.6%)	NA (1)
Nausea and vomiting (2, 1.3%)	-	INVR, self-report validated instrument, English (1) NA (1)
Comfort (1, 0.6%)	-	Interview based on Katherine Kolcaba’s Theory of Comfort, analysed by content analysis
Concerns (1, 0.6%)	-	Semi-structured interview
Constipation and satisfaction with bowel movements (1, 0.6%)	-	Constipation and defecation, Roma III validated criteria, Chinese (1) Satisfaction with bowel movements, 3-degree score, Chinese (1)
Coping (1, 0.6%)	-	CBI, self-report validated questionnaire, Swedish
Cost (1, 0.6%)	-	Mean time to complete and review the protocol
Diarrhoea (1, 0.6%)	-	NA
Fall prevention (1, 0.6%)	-	Behaviour, NOC 3, classification, English
Nutrition (1, 0.6%)	-	NA
Patients’ assessment and care (1, 0.6%)	-	Standard JCI (nursing care is planned within 24 h from admittance, nursing care is tailored using the collected data, nursing plan is updated and modified based on patient reassessment) Standard JCI (patients’ needs are identified based on nursing and medical assessment and they are registered; all patients underwent a screening of pain; the patient is subjected to revaluation in order to determine the response to treatment; the patient is subjected to revaluation in order to plan for continuity of care; the patient is subjected to revaluation at appropriate intervals depending on the treatment plan and identified needs)
Perception of health-related information (1, 0.6%)	-	EORTC QLQ-INFO25, validated questionnaire, Swedish
Physiologic complications (1, 0.6%)	-	Presence of urinary tract infection, respiratory tract infection, skin/mucocutaneous infection, bleeding, febrile state, thrombophlebitis, or pulmonary embolus
Problem solving ability (1, 0.6%)	-	SPSI-R, validated questionnaire, Dutch
Safe medication administration (1, 0.6%)	-	NA
Sleep disturbance (1, 0.6%)	-	NA
Social support (1, 0.6%)	-	SSL, validated questionnaire, Dutch
Survival (1, 0.6%)	-	Time in days from enrolment in the study until death or last date known alive (1)
Tissue integrity (1, 0.6%)	-	Skin and mucous membrane, NOC 3, classification, English
Uncertainty (1, 0.6%)	-	MUIS-C, validated questionnaire, English (1)
Unplanned therapy interruption (1, 0.6%)	-	N of therapy interruptions from the patients’ diary and nurses’ records (1)
Use of sources of information (1, 0.6%)	-	Semi-structured interview (1)

APS POQ: American Pain Society Patient Outcomes Questionnaire; ASK-12: Adherence Starts with Knowledge; BFI: Brief Fatigue Inventory; BPI: Brief Pain Inventory; BPI-C: Brief Pain Inventory—Chinese version; BQ-SF: Barriers Questionnaire—Short Form; CARE-Q: Caring Assessment Instrument; CASC: Comprehensive Assessment of Satisfaction with Care; CBI-24: Caring Behaviours Inventory—24; CES-D: Center for Epidemiological Studies—Depression Scale; CINV: chemotherapy-induced nausea and vomiting; CRI: central venous catheter-related infection; DGSS: Dutch General Self-efficacy Scale; EORTC QLQ-C30: European Organisation for Research and Treatment of Cancer Quality of Life Questionnaire—Cancer 30; EORTC QLQ-C15-PAL: European Organisation for Research and Treatment of Cancer Quality of Life Questionnaire—Core 15—Palliative; ESDS: Enforced Social Dependency Scale; F: female; FACT-F: Functional Assessment of Cancer Therapy Fatigue; FACT-G: Functional Assessment of Cancer Therapy—General; FACT-NH: Functional Assessment of Cancer Therapy-Head and Neck; FACT-NHSI: Functional Assessment of Cancer Therapy-Head and Neck Cancer Symptoms Index; FACIT-Sp: Functional Assessment of Chronic Illness Therapy-Spiritual; GAD-7: Generalized Anxiety Disorder seven-item scale; HADS: Hospital Anxiety and Depression Scale; HCAHPS: Hospital Consumer Assessment of Healthcare Providers and Systems; HCI: Holistic Caring Inventory; ICS: Individualized Care Scale; INVR: Rhodes Index of Nausea, Vomiting and Retching; JCI: Joint Commission International; M: male sex; MDASI: MD Anderson Symptom Inventory; MDASI-NH: MD Anderson Symptom Inventory—Head and Neck; MSAS: Memorial Symptom Assessment Scale; MUIS-C: Mishel Uncertainty in Illness Scale—Community Form; *n*: number of related studies; N: number; NA: not available; NCCN DT: National Comprehensive Cancer Network’s Distress Thermometer; NCSR: National Center for Surveys and Research; NOC: Nursing Outcome Classification; NRS: Numeric Rating Scale; NSC: Nurse Specific Satisfaction with Care; NSO: Nursing-sensitive outcomes; OAG: Oral Assessment Guide; PAM: Patient Activation Measure; PBS: Patient Barriers Survey; PGI: Press Ganey Inpatient survey; PGO: Press Ganey Outpatient survey; PHQ-9: Patient Health Questionnaire; POMS: Profile of Mood States; PPQ: Patient Pain Questionnaire; PASQOC: Patient Satisfaction and Quality in Oncological Care; PSCC: Patient Satisfaction With Cancer Care; PSN-1: Patient Satisfaction With Interpersonal Relationship With Navigator; PSNCQQ: Patient Satisfaction with Nursing Care Quality Questionnaire; PSQ: Patient Satisfaction Questionnaire; PSS: Patient Satisfaction Scale; QONCS: Quality Oncology Nursing Care Scale; QPP: Quality from the Patient’s Perspective; RPS-POQ: Revised American Pain Society—Patient Outcome Questionnaire; SDS: Symptom Distress Scale; SEMCD 6: Self-Efficacy for Managing Chronic Disease Scale; SES: Symptom Experienced Scale; SF-8: Short Form 8 Health Survey; SF-12: Short Form 12-item Health Survey; SF-36: Medical Outcomes Study 36-item Short Form Survey; SMAT: Symptom Management Analysis Tool; SPERC: Stanford Patient Education Research Center; SPSI-R: Social Problem Solving Skills—Revised; SSL: Social Support List; STAI-state: State-Trait Anxiety Scale: state version; USA: United States of America; VAS: Visual Analogue Scale. * For multidisciplinary outcomes, referred also to other healthcare professionals (e.g., nurse assistants, physicians).

**Table 2:** Emerged NSOs (N = 151) classified according to the Oncology Nursing Society (ONS) classification [19].

ONS Outcomes Classification Exemplars	NSOs from Our Scoping Review (N, %)	Detailed ONS Outcomes from Our Scoping Review (*n*, %)
**Symptom control and management**	44 (29.1)	
Pain	10 (6.6)	Resolution/reduction of pain (7, 4.6%), Satisfaction with pain management (2, 1.3%), Knowledge in managing chronic cancer pain (1, 0.6%)
Altered skin or mucous membrane	1 (0.6)	Tissue integrity (1, 0.6%)
Constipation	1 (0.6)	Constipation and satisfaction with bowel movements (1, 0.6%)
Diarrhoea	1 (0.6)	Diarrhoea (1, 0.6%)
Fatigue	3 (2.0)	Fatigue (3, 2.0%)
Insomnia	1 (0.6)	Sleep disturbance (1, 0.6%)
Nausea	2 (1.3)	Nausea and vomiting (2, 1.3%)
Peripheral neuropathy	-	-
Anorexia	-	-
Breathlessness	-	-
Neutropenia	-	-
Other	27 (17.9)	General therapy-related symptoms (8, 5.3%), Barriers and facilitators to intervention adherence/symptoms (5, 3.3%), Therapy-related symptom distress (3, 2.0%), Knowledge in understanding diagnosis and disease management (2, 1.3%), Clinical effectiveness of capecitabine management (1, 0.6%), Comfort (1, 0.6%), Mucositis (1, 0.6%), Oral care situation (1, 0.6%), Health status (3, 2.0%), Physiologic complications (1, 0.6%), Knowledge in medications and side effects (1, 0.6%)
**Economic**	20 (13.2)	
Emergency room visits	4 (2.6)	Emergency department visits (4, 2.6%)
Unexpected readmissions	2 (1.3)	Readmissions/hospitalizations (2, 1.3%)
Length of stay	1 (0.6)	Length of stay (1, 0.6%)
Out-of-pocket costs (family)	-	-
Homecare visits	1 (0.6)	Homecare visits (1, 0.6%)
Costs per day per episode	1 (0.6)	Costs (1, 0.6%)
Other	11 (7.3)	Access to care (2, 1.3%), Timing of care process (4, 2.6%), Clinical appointments (2, 1.3%), Acceptability and accessibility of cancer services (1, 0.6%), Patient disposition at discharge (1, 0.6%), Referrals (1, 0.6%)
**Functional status**	20 (13.2)	
Role functioning	3 (2.0)	Functional status (3, 2.0%)
Ability to carry out usual activities	1 (0.6)	Ability to function independently (1, 0.6%)
Nutritional status	1 (0.6)	Nutrition (1, 0.6%)
Activities of daily living	-	-
Activity tolerance	-	-
Instrumental activities of daily living	-	-
Other	15 (10.0)	Health-related quality of life (8, 5.3%), Self-efficacy (4, 2.6%), Activation (2, 1.3%), Self-care (1, 0.6%)
**Psychological health status**	13 (8.6)	
Anxiety	2 (1.3)	Anxiety (2, 1.3%)
Depression	2 (1.3)	Depression (2, 1.3%)
Spiritual distress	-	-
Coping	1 (0.6)	Coping (1, 0.6%)
Other	8 (5.3)	Psychological distress in general (7, 4.6%), concerns (1, 0.6%)
**Safety (preventable adverse events)**	4 (2.6)	
Infections	1 (0.6)	CRIs (1, 0.6%), Urinary tract infection (1, 0.6%), Respiratory tract infection (1, 0.6%), Skin/mucocutaneous infection (1, 0.6%), Bleeding (1, 0.6%), Febrile state (1, 0.6%), Thrombophlebitis (1, 0.6%), or Pulmonary embolus (1, 0.6%)
Falls	1 (0.6)	Fall prevention (1, 0.6%)
Skin ulcers	-	-
Extravasation incidents	1 (0.6)	Safe medication administration (1, 0.6%)
Hypersensitive reactions	-	-
Other	1 (0.6)	Unplanned therapy interruptions (1, 0.6%)
**-**		
Other *	50 (33.1)	Satisfaction with care received (19, 12.6%), Experiences and perceptions of care received (11, 7.2%), Quality of relationship with nurses (2, 1.3%), Quality of care received (5, 3.3%), Caring and uncaring behaviours (4, 2.6%), Awareness of the importance of received intervention (2, 1.3%), Patients’ assessment and care (1, 0.6%), Perception of health-related information (1, 0.6%), Problem solving ability (1, 0.6%), Social support (1, 0.6%), Survival (1, 0.6%), Uncertainty (1, 0.6%), Use of sources of information (1, 0.6%)

CRIs: central venous catheter-related infections; *n*: number. * Domain added by the authors. In bold, name of ONS’s outcome categories.

## Supplementary Materials

The following supporting information can be downloaded at: https://www.mdpi.com/article/10.3390/nursrep13030096/s1, Table S1: Preferred Reporting Items for Systematic Reviews and Meta-Analyses Extension for Scoping Reviews (PRISMA-ScR) Checklist; Table S2: Abstract reporting checklist required from the Preferred Reporting Items for Systematic Reviews and Meta-Analyses Extension for Scoping Reviews (PRISMA-ScR); Table S3: Data extraction from the included studies; Table S4: Summary of NSOs and characteristics from the available studies. Ref [16,18,21,22,23,24,25,26,27,28,29,30,31,32,33,34,40,43,45,46,47,48,49,50,51,52,53,54,55,56,57,58,59,60,61,62,63,64,65,66,67,68,69,76,83,86,87,88,89,90,91,92,93,94,95,96,97,98,99,100,101,102,103] are cited in the supplementary.

## Public Involvement Statement

There was no public involvement in any aspect of this research.

## Guidelines and Standards Statement

This manuscript was drafted against the *Preferred Reporting Items for Systematic Reviews and Meta-analysis Extension for Scoping Review* statement [40].
