# Supplementary material for: What Nursing-Sensitive Outcomes Have Been Investigated to Date among Patients with Solid and Hematological Malignancies? A Scoping Review
Source: Nurs Rep. 2023 Aug 15;13(3):1101–25. doi: 10.3390/nursrep13030096 (PMC10443292; doi:10.3390/nursrep13030096)
Supplement: Supplementary file 1 [file nursrep-13-00096-s001.zip › nursrep-2525707-supplementary.pdf]

Table S1. Preferred Reporting Items for Systematic Reviews and Meta-Analyses Extension for Scoping Reviews (PRISMA-ScR) Checklist [40].

SECTION AND TOPIC	ITEM #	PRISMA-ScR CHECKLIST ITEM	Location where item is reported #
**Title**			
Title	1	Identify the report as a scoping review.	Title
**Abstract**			
Structured summary	2	See the PRISMA 2020 for Abstracts checklist.	Supplementary table 2
**Introduction**			
Rationale	3	Describe the rationale for the review in the context of what is already known. Explain why the review questions/objectives lend themselves to a scoping review approach.	Paragraph 1
Objectives	4	Provide an explicit statement of the questions and objectives being addressed with reference to their key elements (e.g., population or participants, concepts, and context) or other relevant key elements used to conceptualize the review questions and/or objectives.	Paragraphs 2.1, 2.2, and 2.3
**Methods**			
Protocol and registration	5	a) Provide registration information for the review, including register name and registration number, or state that the review was not registered.<br>b) Indicate where the review protocol can be accessed, or state that a protocol was not prepared.<br>c) Describe and explain any amendments to information provided at registration or in the protocol.	NA
Eligibility criteria	6	Specify characteristics of the sources of evidence used as eligibility criteria (e.g., years considered, language, and publication status), and provide a rationale.	Paragraph 2.5
Information sources*	7	Describe all information sources in the search (e.g., databases with dates of coverage and contact with authors to identify additional sources), as well as the date the most recent search was executed.	Paragraph 2.4
Search	8	Present the full search strategies for all databases, registers and websites, including any filters and limits used.	Fig. 1
Selection of sources of evidence†	9	Specify the methods used to decide whether a study met the inclusion criteria of the review, including how many reviewers screened each record and each report retrieved, whether they worked independently, and if applicable, details of automation tools used in the process.	Paragraph 2.5
Data charting process‡	10	a) List and define all outcomes for which data were sought. Specify whether all results that were compatible with each outcome domain in each study were sought (e.g. for all measures, time points, analyses), and if not, the methods used to decide which results to collect.<br>b) List and define all other variables for which data were sought (e.g. participant and intervention characteristics, funding sources). Describe any assumptions made about any missing or unclear information	Paragraphs 2.3 and 2.6
Data items	11	List and define all variables for which data were sought and any assumptions and simplifications made.	NA
Critical appraisal of individual sources of evidence§	12	If done, provide a rationale for conducting a critical appraisal of included sources of evidence; describe the methods used and how this information was used in any data synthesis (if appropriate).	NA
Synthesis of results	13	Describe the methods of handling and summarizing the data that were charted.	Paragraph 2.7
**Results**			
Selection of sources of evidence	14	a) Describe the results of the search and selection process, from the number of records identified in the search to the number of studies included in the review, ideally using a flow diagram.	Figure 1, Paragraph 3.1

		b) Cite studies that might appear to meet the inclusion criteria, but which were excluded, and explain why they were excluded.	
Characteristics of sources of evidence	15	For each source of evidence, present characteristics for which data were charted and provide the citations.	Paragraph 3.1, Supplementary Table 3
Critical appraisal within sources of evidence	16	If done, present data on critical appraisal of included sources of evidence (see item 12).	NA
Results of individual sources of evidence	17	For each included source of evidence, present the relevant data that were charted that relate to the review questions and objectives.	Paragraphs 3.1, 3.2, and 3.6, Table 1, Table 2, Supplementary Table 3 and Supplementary Table 4
Synthesis of results	18	Summarize and/or present the charting results as they relate to the review questions and objectives.	Paragraphs 3.1, 3.2, and 3.3, Table 1, Table 2, Supplementary Table 3, Supplementary Table 4
**Discussion**			
Summary of evidence	19	Summarize the main results (including an overview of concepts, themes, and types of evidence available), link to the review questions and objectives, and consider the relevance to key groups.	Paragraph 4.1, 4.2, and 4.3
Limitations	20	Paragraph	Paragraph 4.4
Conclusions	21	Provide a general interpretation of the results with respect to the review questions and objectives, as well as potential implications and/or next steps.	Paragraph 5
**Other information**			
Funding	22	Declare any competing interests of review authors.	Conflicts of interest

NA: not applicable.

Table S2. Abstract reporting checklist required from the Preferred Reporting Items for Systematic Reviews and Meta-Analyses Extension for Scoping Reviews (PRISMA-ScR) [40].

SECTION AND TOPIC	ITEM #	PRISMA-ScR CHECKLIST ITEM	Location where item is reported #
**Title**			
Title	1	Identify the report as a scoping review.	Text of abstract
**Background**			
Objectives	O	Provide an explicit statement of the main objective(s) or question(s) the review addresses.	Text of abstract
**Methods**			
Eligibility criteria	3	Specify the inclusion and exclusion criteria for the review.	Text of abstract
Information sources	4	Specify the information sources (e.g. databases, registers) used to identify studies and the date when each was last searched.	Text of abstract
Risk of bias	5	Specify the methods used to assess risk of bias in the included studies.	NA
Synthesis of results	6	Specify the methods used to present and synthetize results.	Text of abstract
**Results**			
Included studies	7	Give the total number of included studies and participants and summarize relevant characteristics of studies.	Text of abstract
Synthesis of results	8	Present results for main outcomes, preferably indicating the number of included studies and participants for each. If meta-analysis was done, report the summary estimate and confidence/credible interval. If comparing groups, indicate the direction of the effect (i.e. which group is favored).	Text of abstract
**Discussion**			
Limitations of evidence	9	Provide a brief summary of the limitations of the evidence included in the review (e.g. study risk of bias, inconsistency and imprecision).	Text of abstract
Interpretation	10	Provide a general interpretation of the results and important implications.	Text of abstract
**Other**			
Funding	11	Specify the primary source of funding for the review.	NA
Registration	12	Provide the register name and registration number.	NA

NA: not applicable.

Table S3. Data extraction from the included studies (n = 60).

First author, year, country	Purpose(s) of the study	Study design, sampling, and duration	Setting and/or units involved	Studied population (N, mean age ± SD, prevailing gender, primary tumour)	Nursing care studied intervention(s)	Measured outcomes (measures/metrics used, data collection source, timing of detection)	Main results
Andersson et al. (1999) [67], Sweden	1. To evaluate oral status in a group of patients with haematological malignancies and undergoing chemotherapy treatment using OAG<br><br>2. To test the reliability of the OAG	Pilot assessment study<br><br>Convenience<br><br>From September 1994 to May 1996	The haematological section of the Department of Internal Medicine at a hospital in southern Sweden	N = 16 with an haematological cancer<br>Mean age = 60.7 years<br>M = 13 (81.2%)<br>N = 7 acute leukaemia<br>N = 7 non-Hodgkin's lymphoma-a<br>N = 7 Hodgkin's disease<br>N = 7 myeloma	Daily assessment of oral status using OAG	Patients' oral care situation (OAG, assessment)*<br>Daily 5 times a week	All patients reported varying degrees of alterations in the oral cavity, especially in the mucous membranes, teeth/dentures and gums.<br><br>The inter-rater agreement between the nurses and the dental hygienist was good for saliva and swallow, and moderate for voice and gums. The OAG seems to be a reliable and clinical useful tool
Ayaad et al. (2019) [32], Jordan	1. To evaluate nursing care<br><br>2. To determine patient and family's engagement in patient care	Two-group post-test design study<br><br>Stratified random<br><br>NA	Four adult inpatient oncology units at King Hussein Cancer Center	N = 169 with cancer hospitalized in the target units for at least 3 days<br>Mean age = NA<br>F = 90 (53.3%)<br>N = 89 (control group)<br>40–50 years: 26 (29.2%)<br>F = 46 (51.7%)<br>N = 80 (experimental group)<br>50–60 years: 30 (37.5%)<br>F = 44 (55.0%)<br>NA	Structured nurse leader rounds with a scripted nurse leader tool (study group)<br><br>Unstructured nurse leader rounds performed by acting nurse leader (control group)	Patient satisfaction with nursing care (PSNCQQ, validated questionnaire)<br><br>T1 = within 5 days after discharge	There was a significant difference between the groups (t = -9.213, p = 0.001) regarding patients' satisfaction with nurses' concern and caring. Structured nurse leader rounds have a significant impact on enhancing patient satisfaction with the patient engagement process
Bellomo (2016) [52], USA	1. To examine if tailored nurse coaching	Two-stage study of mixed-methods	Intermountain – Southwest Cancer	N = 24 with various types of cancer<br>NA	Patient-centred assessment and education protocol	Barrier to or facilitators of the patient's adherence, symptoms	Based on the responses to the ASK-12 tool, all patients reported that

	interventions in addition to patient education are more effective than standard chemotherapy education alone in improving symptom management and patient adherence in adult cancer patients receiving oral chemotherapy agents	Convenience<br><br>3 months	Center of Cedar City Hospital	NA<br>NA	utilizing the MASCC Teaching Tool for Patients Receiving Oral Agents for Cancer and tailored follow-up telephone calls by an oncology nurse navigator	and side effects (ASK-12, validated tool)<br>T1 = weekly for the first month after initiating treatment, T2 = twice a month for the second and third months, T3 = monthly thereafter for the course of treatment	the education program, the individualized written information, and the follow-up telephone contact reinforced their knowledge and understanding of their oral chemotherapy as well as enhanced their comfort level in being able to manage and adhere to the treatment
Blackburn et al. (2019) [23], USA	1. To identify easy-to-implement complementary therapies that can supplement pharmacological pain management for cancer patients	A pre-post trial of a quality project<br><br>Convenience<br><br>2017	Inpatient and ambulatory oncology units at the James Cancer Hospital in Ohio	N = 242 with cancer pain<br>NA<br>NA<br>NA	'Comfort kits' (handheld massagers, guided imagery audiotapes, aromatherapy essential oils, sleep masks) prepared by pain clinical nurse specialists	Patients' satisfaction with pain management (non-declared scale for ambulatory patients and two statements for inpatients)<br>T0, T1 = +24 hours after use<br><br>Patients' pain (10 degree not-declared scale)<br>T0, T1 = +24 hours after use	During the trial, pain intensity decreased by 2.25 points after the use of the comfort kit. 63% of the inpatient units reported an increase in patient satisfaction with pain management (the average increase in percentile ranking was 16 points). In the ambulatory setting, 57% of the ambulatory units reported an increased patient satisfaction when comparing their baseline (the average increase in percentile ranking was 38 points). Patients also showed an overall decrease in the use of pharmacologic pain interventions and an increase in ambulation in the 24-hour period after implementation
Booth et al. (2005) [25], UK	1. To explore number and content of patients' concerns,	Prospective study	Two specialist gynaecological oncology centres	N = 70 with a first-time diagnosis of a gynaecological cancer	Initial clinical nurse specialist contact	Number of patients' concerns	Women who had contact with a clinical nurse specialist at the

	psychological distress and use of sources of information	Convenience<br><br>NA	in the Northwest of England	Mean age = 52 years<br>F = 70 (100.0%)<br>100.0% = gynaecological cancer		(semi-structured interview)<br>T0, T1 = +6 month from diagnosis<br><br>Use of sources of information (semi-structured interview)<br>T0, T1 = +6 month from diagnosis<br><br>Patients' psychological distress (HADS, self-report validated questionnaire)<br>T0, T1 = +6 month from diagnosis	time of diagnosis were significantly less likely to have four or more significant worries relating to their illness and experienced a clinically significant reduction in the level of psychological distress. As contact with the hospitals decreased, there was a significant reduction in the use of professional sources of information and a significant increase in the use of non-professional sources. Women who have maintained contact with the clinical nurse specialist showed a slight non-significant decrease in the use of the specialist nurse for information
Braamse et al. (2010) [28], the Netherlands	1. To evaluate the outcome of stepped care for psychological distress on functional status and other aspects of quality of life in patients with haematological malignancy treated with autologous HSCT	Protocol of a multicentre RCT<br><br>Permutated-blocked randomization concealment with stratification<br><br>43 months	One of the large Dutch university hospitals and one Dutch teaching hospital	N = 286 expected patients receiving autologous HSCT following high-dose chemotherapy<br>Not applicable<br>Not applicable<br>100.0% = haematological malignancies	Web-based stepped care intervention (study group)<br><br>Usual care (control group)	Psychological distress (HADS, validated self-report questionnaire, score ≥ 8 or PHQ-9, self-report questionnaire, score ≥ 10 or STAI-state adapted, self-report validated questionnaire, score ≥ 40)<br>T1 = +13 weeks post HSCT, T2 = +30 weeks post HSCT, T3 = +42 weeks post HSCT<br><br>Functional status: QOL (EORTC-QLQ-C30, validated questionnaire	Not appropriate

						or SF-36, validated questionnaire), belief in ability to function independently (DGSS, validated questionnaire), problem solving skills (SPSI-R, validated questionnaire), interactions and discrepancies in receiving social support (SSL, validated questionnaire)<br>T0, T1 = +13 weeks post HSCT, T2 = + 30 weeks post HSCT,<br><br>Care evaluation (GGZ-thermometer, validated questionnaire)<br>T3 = +42 weeks post HSCT	
Chang et al. (2005) [86], Taiwan	1. To explore differences in perceived importance of nursing caring behaviours between patients with cancer pain and oncology nurses<br><br>2. To explore the relationship between level of pain intensity and importance of various nursing caring behaviours	Cross-sectional and descriptive correlational study<br><br>Consecutive<br><br>NA	Three regional hospitals with oncology units in northern Taiwan	N = 100<br>Mean age = 41.4 years<br>F = 65 (65.0%)<br><br>N = 50 patients diagnosed with cancer experiencing pain in the last 24 hours and receiving pain medication<br><br>Mean age = 57.70 ± 13.2 years<br>M = 35 (70%)<br>N = 10 lung cancer<br>N = 7 hepatoma<br>N = 4 breast cancer<br>N = 4 rectal cancer<br>N = 4 nasopharyngeal carcinoma	Nursing caring behaviours	Patients' pain intensity (BPI-C, self-report questionnaire, filled out by patients and nurses)<br>T1 = +3 days from admission<br><br>Caring behaviour (CARE-Q, validated assessment tools, filled out by patients and nurses)<br>T1 = +3 days from admission	Patients ranked 'being accessible', 'monitors and follows through' and 'anticipates' as being the most important nursing caring behaviours; nursing staff ranked 'being accessible', 'explains and facilitates', and 'monitors and follows through' as being the most important behaviours. The patients self-reported level of pain was significantly positively correlated with the patient rating of 'anticipates'

				N = 21 others<br><br>N = 50 nurses who had provided primary care to the eligible patients for three days<br>Mean age = 25 ± 2.57 years<br>F = 50 (100.0%)<br>Not applicable			behaviour. Patient self-reported level of pain interference was significantly positively correlated with the 'monitors and follows through' behaviour and negatively correlated with the 'explains and facilitates' behaviour. Staff perception of both a patient's level of pain intensity and pain interference was significantly positively correlated with staff rating of the 'being accessible' behaviour
Charalambous (2013) [56], Cyprus	1. To assess patient satisfaction with the nursing care provided	Multi-site descriptive study<br><br>Simple random<br><br>From February 2010 to January 2011	Three specialized oncology centres in Cyprus	N = 272 first time patients with cancer<br>Mean age = 55 ± 1.4 years<br>F = 139 (51.1%)<br>66.2% breast and prostate cancer<br>17.3% = head and neck cancer<br>16.5% = lung cancer	Nursing care	Patient satisfaction with nursing care (PSS, self-report validated questionnaire)<br>T1 = + 1 week after the discharge<br><br>Dimensions of care received (7 single questions adapted by the 10-care dimension by NCSR)<br>T1 = + 1 week after the discharge	Participants were overall satisfied by the nursing care (mean 3.5). Socio-demographic and clinical variables can positively or negatively influence the satisfaction levels. However, dissatisfaction was expressed in relation to the 7-care dimensions regarding hospital management as well as pain and discomfort. Overall, the patients appeared more satisfied with the interpersonal-trusting aspects of nursing care rather that the technical–professional or the interpersonal–educational aspects

Coleman et al. (2010) [30], USA	1. To compare certified nurses with non-certified nurses for knowledge and clinical behaviours related to symptom management of pain and CINV, patient satisfaction, and nurse satisfaction	Prospective and descriptive study<br><br>NA<br><br>NA	Two inpatient oncology units, two outpatient oncology clinics, and two infusion centres at an Academic Health Science Centre in the southern USA	N = 343<br>Mean age = NA<br>F = 185 (53.9%)<br>N = 35 certified oncology nurses<br>41–50 years: 14 (40%)<br>F = 31 (89%)<br>N = 58 non-certified oncology nurses<br>51–60 years: 18 (31%)<br>F = 52 (90%)<br>N = 270 cancer patients<br>Mean age = 56 years<br>M = 54.8%<br>NA	Comparison between certified and non-certified oncology nurses for knowledge and clinical behaviours related to symptom management of pain and CINV	Patients' knowledge and experiences in managing chronic cancer pain (PPQ, validated questionnaire)<br><br>Patients' experiences with CINV (INVR, self-report validated instrument)<br><br>Patient satisfaction with main management (three scales from the PGI)<br><br>Nurses' expertise on pain management (NKASRP, validated survey)<br><br>Nurses' knowledge and attitudes about the management of CINV (Nausea Management: Nurses' Knowledge and Attitudes Survey, investigator-developed survey)<br><br>Nurses' job satisfaction (adapted tool)	Certified nurses scored higher than non-certified nurses on the NKASRP as well as the Nausea Management: Nurses' Knowledge and Attitudes Survey. The chart audits showed that certified nurses followed National Comprehensive Cancer Network guidelines for CINV management more often than non-certified nurses. Job satisfaction is fairly high for oncology nurses and patient satisfaction is high
Coolbrandt et al. (2018) [65], Belgium	1. To describe a step-by-step overview of the development of a nursing intervention aimed at reducing chemotherapy-related symptom burden<br><br>2. To describe the actual intervention,	Protocol of a mixed-method study of a complex intervention<br><br>NA<br><br>NA	Home	NA<br>Not applicable<br>Not applicable	Development and implementation of a nursing intervention to reduce chemotherapy-related symptom burden (CHEMO-SUPPORT) following the Intervention Mapping Approach	Patients' satisfaction (open questions and semi-structured interviews)<br><br>Symptom distress (NA)<br>NA<br><br>Symptom severity (NA)<br>NA	Not appropriate

	as it will be implemented and studied					Self-efficacy (NA)<br>NA<br><br>Outcome expectations (NA)<br>NA<br><br>Self-care (NA)<br>NA	
Craig et al. (1999) [87],<br>UK	1. To study the impact on quality of care of a dedicated haematology nurse who would coordinate and deliver blood product and related treatments both in the community and at home	Prospective study<br><br>Convenience<br><br>12 months	A hospital haematology unit and participants' homes	N = 45 with haematological disorders requiring regular transfusion<br>Mean age = 71 years<br>F = 28 (62.2%)<br>N = 9 myelodysplastic syndrome<br>N = 3 acute myeloid leukaemia<br>N = 4 myeloma<br>N = 7 non-Hodgkin's lymphoma<br>N = 2 Hodgkin's disease<br>N = 3 chronic lymphatic leukaemia<br>N = 5 chronic myeloid leukaemia<br>N = 12 non-haematological neoplasms	Dedicated haematology nurse to manage provision of blood product support in a hospital haematology unit and at home	Waiting time from admission and total length of patient stay (patient questionnaires and nurse observation to document waiting times)<br>During the study period<br><br>Patients' perceptions of importance of the process (ranking questionnaire, 5-point scale)<br>NA<br><br>Patient satisfaction with the process (ranking questionnaire, 5-point scale)<br>NA	The nurse-led service resulted in a significant reduction in the waiting time from admission to transfusion ($p = 0.003$) and in the total length of in-patient stay ($p = 0.037$). The importance of and satisfaction with different aspects of the care of the transfusion process showed improved satisfaction scores for all aspects of care.<br>Preference for home blood sampling instead of hospital increased from 24% before to 100% after intervention
Curcio et al. (2012) [54],<br>USA	1. To implement and evaluate a survivorship protocol for cancer survivors to improve their knowledge about their cancer and decrease their anxiety	Pre/post-test design study<br><br>Convenience<br><br>NA	A clinic in a small community cancer centre in the south-eastern USA	N = 30 survivors<br>Mean age = 64 ± 13 years<br>F = 25 (83.3%)<br>N = 16 breast cancer<br>N = 8 haematological neoplasms<br>N = 3 lung cancer<br>N = 3 gastrointestinal cancer	Individualized survivorship protocol	Participants' knowledge (Knowledge questionnaire derived from Miller, 2008)<br>T0, T1 = + 1 month after the protocol visit<br><br>Participant anxiety (GAD-7, validated self-report questionnaire)	One month after the survivorship protocol was delivered, knowledge about diagnosis, treatments, recommended follow-up, signs of recurrence, and late side effects increased.<br>Anxiety scores were lower one month after

	2. To evaluate the satisfaction of survivors, staff, and PCPs with the program<br><br>3. To assess fidelity to evidence-based follow-up recommendations<br><br>4. To analyse the costs and benefits of offering the service					T0 and T1 = one month after the protocol visit<br><br>Satisfaction with protocol (satisfaction survey)<br>T1 = at the end of the study<br><br>Fidelity to evidence-based follow-up recommendations (chart review and participants' interviews)<br>T1 = +1 month after the protocol visit<br><br>Cost (mean time to complete and review the protocol)<br>NA	the intervention, and satisfaction with the protocol was high.<br>The protocol is a feasible method of educating cancer survivors; however, data regarding costs were not available
De Veer et al. (2020) [64], the Netherlands	1. To gain an understanding of how participants evaluate a self-management support intervention with an integrated eHealth application<br><br>2. To examine the possible effects of the intervention on patient activation and QOL	Pre/post-test mixed-method study<br><br>Convenience<br><br>From November 2016 to May 2018	Home setting	N = 36 with an incurable form of cancer, new referral for a continuity home visit<br>61–70 years: 14 (40.0%)<br>F = 18 (50.0%)<br>NA	Structured self-management support intervention	Perceived application of the intervention (11-point Likert-type scale, self-report questionnaire)<br>T1 = + 12 weeks<br><br>Patients' satisfaction with nurse led model ('5 A's model', self-report questionnaire + 11-point Likert-type scale, self-report questionnaire)<br>T1 = + 12 weeks<br><br>Patients' activation (PAM, validated self-report questionnaire)<br>T0, T1 = + 12 weeks	74% of patients referred that the nurse applied the '5 A's model' in full.<br>85% of patients were satisfied with the assessment of their needs and the advice received by the nurses.<br>Patients perceived nurses' expertise, empathy, and time for themselves as a pleasure, and appreciated that visits were carried out at their homes.<br>No statistically significant changes in patient activation and QOL

						QOL (EORTC QLQ-C15-PAL, validated questionnaire)<br>T0, T1 = + 12 weeks	
Du Pen et al. (2000) [53], USA	1. To test the application of the Cancer Pain Algorithm with community oncologists and nurses	RCT<br><br>Clustered stratified random<br><br>From January 1996 to June 1997	Nine institutions of Puget Sound clinics (two managed care organisations, three small community hospitals, four large community or regional hospitals)	N = 105 ambulatory patients with diagnostic evidence of locally invasive or metastatic solid tumours with at least a 6-month life expectancy<br>Mean age = 61 years<br>F = 64 (60.9%)<br>N = 54 (experimental group)<br>Mean age = 61 ±1.5 years<br>F = 72.0%<br>N = 51 (control group)<br>Mean age = 61 ± 1.4 years<br>M = 52.0%<br>N = 22 lung cancer<br>N = 21 breast cancer<br>N = 12 colorectal cancer<br>N = 9 ovarian cancer<br>N = 9 multiple myeloma<br>N = 32 others	Care by algorithm-trained practitioners (study group)<br><br>Care by non-algorithm-trained practitioners (control group)	Patients' pain (BPI, validated questionnaire) *<br>T0, T1 = + 1 month, T2 = + 2.5 months, T3 = + 4 months<br><br>Patients' symptoms (MSAS, validated questionnaire) *<br>T0, T1 = + 1 month, T2 = + 2.5 months, T3 = + 4 months<br><br>Patients' barriers (PBS, validated questionnaire) *<br>T0, T1 = + 1 month, T2 = + 2.5 months, T3 = + 4 months	Patients of trained providers reported a significant reduction in usual pain over the four months of data collection compared with patients of untrained providers (t = 2.0; p = 0.05).<br>Patients with higher mean opioid adherence experienced larger drops in symptom distress on the MSAS (r = -0.30, p = 0.003), while patients' adherence scores were not significantly related to patients' scores on the PBS
Ekwall et al. (2003) [45], Sweden	1. To describe what women diagnosed with primary gynaecological cancer reported to be important during their interaction within the healthcare system	Qualitative study<br><br>Consecutive<br><br>From fall 1996 to spring 1997	A specialized gynaecological oncology care unit at a university hospital in central Sweden	N = 14 at first diagnosis with gynaecological cancer<br>Mean age = 57.5 years<br>F = 14 (100.0%)<br>N = 2 cervical cancer<br>N = 4 ovarian cancer<br>N = 8 uterine cancer	-	Patients' experiences with the healthcare system and how illness affects daily life (semi-structured interview analysed using a qualitative content analysis)*<br>T1 = NA	Three partly overlapping categories (optimal care, good communication, and self-image and sexuality) were found to be of central importance in quality of health care. Information and everyday conversation were of great

							significance. Participants stated that health care should provide individualized information and care to satisfy their individual needs and reinforce their self-image. Nurses have an important role in strengthening women's feelings of hope and supporting them in maintaining as positive a self-image as possible
Ferreira et al. (2017) [88], Brazil	1. To monitor the adverse effects of antineoplastic chemotherapy in patients undergoing outpatient treatment<br><br>2. To describe the telephone follow-up as a strategy to provide comfort	Longitudinal, prospective study with quantitative–qualitative approach<br><br>Convenience<br><br>From October 2012 to February 2013	Chemotherapy Outpatient Clinic in the Oncology Centre of the University Hospital of Brasília	N = 21 diagnosed with some form of malignant neoplasm<br>Age: 30–78 years<br>NA<br>NA	Weekly nursing telephone consultation	Chemotherapy's signs and symptoms of adverse effects (checklist format used based on scientific literature)<br>During the consultations<br><br>Patients' comfort (interview based on Katherine Kolcaba's Theory of Comfort, analysed by content analysis)<br>During the consultations	All participants reported satisfaction with the telephone follow-up received. Obtaining an accurate view of the adverse reactions experienced is of great relevance for dose changes, supportive care, and education that should be directed to the patient. The telephone contact consists of an adequate and important comfort strategy during the patient's follow-up led by the nursing team
Fukui et al. (2011) [29], Japan	1. To investigate whether a CST program in breaking bad news for nurses would be useful for improving QOL of patients and improving satisfaction with healthcare	Secondary analysis of an RCT<br><br>Random and consecutive<br><br>From January to December 2006	Four cancer screening institutions in east Japan	N = 86 newly diagnosed<br>Mean age = 61.1 years<br>F = 52 (60.5%)<br>N = 41 (study group)<br>Mean age = 61.4 ± 10.8 years<br>F = 25 (61.0%)<br>N = 45 (control group)	One-on-one psychological and informational support after nurses' attendance of the CST program	Patients' QOL (SF-8, validated questionnaire)<br>T1 = +1-week after cancer diagnosis, T2 = + 1-month after cancer diagnosis, T3 = + 3-month after cancer diagnosis	Analysis of variance revealed significant between-group differences: the experimental group showed significant increase of the mental aspects of SF-8 ($F = 3.48$; $p = 0.03$) and of

	professionals when informed of their cancer diagnosis just after a cancer screening test			Mean age = 60.9 ±14.3 years<br>F = 27 (60.0%)<br>N = 40 gastric cancer<br>N = 24 colorectal cancer<br>N = 22 breast cancer		Patients' satisfaction (VAS, scale)<br>T1 = +1-week after cancer diagnosis, T2 = + 1-month after cancer diagnosis, T3 = + 3-month after cancer diagnosis	satisfaction with the nurse (F = 3.18; *p* = 0.04)
Given et al. (2002) [50], USA	1. To compare data from a 20-week supportive nursing intervention plus conventional care versus conventional care alone among patients undergoing an initial course of chemotherapy and reported pain and fatigue at baseline in numbers of symptoms, patient's functioning, and reduction of pain and fatigue	Pre/post-test study<br><br>Simple random<br><br>32 weeks	Four outpatient cancer treatment sites (two affiliated with comprehensive cancer centre and two community cancer treatment clinics)	N = 113 with cancer undergoing an initial course of chemotherapy<br>Mean age = 57.9 years<br>F = 81 (71.7%)<br>N = 53 (study group)<br>Mean age = 59 ± 10 years<br>F = 38 (72.0%)<br>N = 60 (control group)<br>Mean age = 57 ±11 years<br>F = 43 (72.0%)<br>N = 39 breast cancer<br>N = 31 gynaecological cancer<br>N = 26 lung cancer<br>N = 16 colon cancer<br>N = 1 lymphoma	Cognitive behavioural nursing supportive care intervention and conventional care (study group)<br><br>Conventional care (control group)	Number of symptoms (SES, 5-point scale, and telephone-based interview)<br>T0, T1 = + 10 weeks, T2 = + 20 weeks<br><br>Patients' functioning (two subscales from the SF-36 and telephone-based interview)<br>T0, T1 = + 10 weeks, T2 = + 20 weeks	At T2, patients in the study group reported fewer symptoms and improved impact on their physical and social role functioning.<br>The total resolution of pain and fatigue was in the expected direction (for example, the specialized intervention improved reports of both pain and fatigue over time) although not statistically significant
Godino et al. (2006) [27], Spain	1. To evaluate fatigue levels and severity in patients diagnosed with colon or gastric cancer before, during, and after chemotherapy treatment<br><br>2. To measure the degree of patients' satisfaction with a	Pre/post-test study<br><br>Simple random<br><br>From July 2001 to September 2002	Colorectal and digestive tumours unit of a comprehensive cancer centre in Barcelona	N = 40 with colorectal or gastric cancers<br>Mean age = 60.3 years<br>M = 21 (52.5%)<br>N = 23 (experimental group)<br>Mean age 58.5 ± 11.34 years<br>M = 12 (52.2%)<br>N = 17 (control group)<br>Mean age = 62.7 ±8.8<br>M = 9 (52.9%)	Individualised and structured patient education program to manage fatigue	Patients' fatigue levels (FACT-F, validated scale)<br>T0, T1 = second cycle of chemotherapy treatment, T2 = +1 month after the completion of treatment (study group)<br>T0, T2 = +1 month after the completion of treatment (control group)	After the nursing intervention there was a decrease in the levels of fatigue in the experimental group, whereas the group of patients that did not receive this intervention showed an increase in fatigue levels during the treatment. The nursing intervention with the individualised education and

	nursing intervention aimed at decreasing fatigue					Patient satisfaction with nursing intervention (satisfaction questionnaire, previously piloted self-report questionnaire)<br>T1 = +1 month after the completion of treatment (study group)	counselling has provided patients with cancer with an effective tool to manage fatigue
Gordils-Perez et al. (2017) [43], USA	1. To evaluate the effect of the oncology nurse navigator program on access to care, patient and provider satisfaction, and patient referrals to clinical trial	Descriptive cohort study with historic control<br><br>Consecutive<br><br>From March 2015 to December 2015	Rutgers Cancer Institute of New Jersey in New Brunswick	N = 289<br>Mean age = 60.4 years<br>F = 194 (67.1%)<br>N = 125 patients with a new gynaecological cancer diagnosis<br>N = 93 (study group)<br>N = 32 (historic control)<br>Mean age = 61.0 years<br>F = 100.0%<br>N = 56 endometrial cancer<br>N = 37 ovarian cancer<br>N = 17 cervical cancer<br>N = 4 Fallopian tube cancer<br>N = 5 other<br>N = 164 patients with a new diagnosis of haematological malignancy<br>N = 89 (study group)<br>N = 75 (historic control)<br>Mean age = 60.0 years<br>M = 58.0%<br>N = 59 non-Hodgkin lymphoma<br>N = 36 multiple myeloma	Oncology nurse navigation program	Access to care (time for the first patient call to first oncology provider consultation)<br><br>Patient satisfaction with relationship with navigator (PSN-1, validated scale)<br>T1 = post implementation<br><br>Patient satisfaction with cancer care (PSCC, validated scale)<br>T1 = post implementation	A significant decrease in the mean days from first provider visit to first therapy was observed in the haematology population. In both groups, time from contact to first visit and from first visit to initiation of treatment decreased. Patient satisfaction with the oncology nurse navigators was uniformly high in both populations. Patients were also highly satisfied with the cancer care they received. Providers were highly satisfied with the program, and the navigation program did not increase clinical trials enrolment

				N = 16 Hodgkin lymphoma<br>N = 23 acute myeloid leukaemia<br>N = 14 acute lymphoblastic leukaemia<br>N = 16 other			
Gray et al. (2002) [33],<br>Canada	1. To assess the role of physicians and nurses in providing supportive care to breast cancer patients	Retrospective study<br><br>Simple random<br><br>NA	Ontario Cancer Registry	N = 731<br>Age < 65 years = 72.0%<br><br>F = 731 (100.0%)<br><br>Invasive breast cancer	Supportive care	Patients' perceptions of supportive care involvement of physicians and nurses (survey questionnaire, non-validated) *<br>T1 = 23–36 months after cancer diagnosis	45% reported having talked with a nurse about one or more supportive care issues. Most of the women who sought supportive care help from physicians and nurses found their contact helpful. Seeking supportive care from a nurse was associated with younger age, working status, higher education, additional health insurance, higher household income, and receiving chemotherapy
Griffiths et al. (2012) [21],<br>UK	1. To identify patient outcomes sensitive to the quality of nursing services to guide the development of an outcomes-based quality measurement system	Scoping review<br><br>NA<br><br>2002-2011	Ambulatory cancer chemotherapy centres	28 studies<br>NA<br>NA<br>NA	Communication skills training, communication skills<br><br>Exercise and additional psychosocial intervention<br><br>Pre-assessment, targeted screening, structured follow-up evidence-based practice protocol,	Communication and knowledge (changes in professional behaviour, NA)<br>NA<br><br>Fatigue (NA)<br>NA<br><br>Nausea and vomiting (NA)<br>NA	A broad range of outcomes potentially sensitive to nursing service quality in ambulatory cancer chemotherapy (patient experience, nausea, vomiting, mucositis, and safe medication administration) emerged from individual trials; however, relatively little clear evidence of effect on outcomes derived from systematic reviews and no evidence

					provision of advice on self-care		associating characteristics of nursing services with outcomes were found
					Nutritional supplementation and counselling	Nutrition (NA)<br>NA	
					Good oral care, client education nurse-led or based on nurses' advice	Oral mucositis (NA)<br>NA	
					Pain management guidelines, patient training, information giving, psychological interventions	Pain (progression or reduction, NA)<br>NA	
					Multifaceted nurse-led programme	Safe medication administration (NA)<br>NA	
					Patient education, self-care, appropriate support and assessment	Septicaemia (rates of infections)<br>NA	
					Distress, education, counselling, cognitive behaviour therapy, mindfulness-based stress reduction, expressive writing	Wellbeing and function (depression and anxiety, NA; sleep disturbance, NA)<br>NA	
					NA	Patient experience (NA)<br>NA	
					NA	Diarrhoea (NA)<br>NA	
Halldòrsdottir and Hamrin (1997) [59], Iceland	1. To explore the essential structure of caring and uncaring encounters with nurses and other health professionals	Phenomenological qualitative study<br><br>Purposive	NA	N = 9 in remission or recovery phase of cancer<br>Age: 41–72 years<br>F = 5 (55.5%)	Caring and uncaring encounters with nurses and other health professionals	Patients' perceptions of the encounters (in-depth dialogues analysed by thematic analysis) *	Three main categories emerged as structure of a caring encounter: nurse/health professional is perceived as being competent,

		NA		NA = carcinoid cancer<br>NA = breast cancer<br>NA = colon cancer<br>NA = uterine cancer<br>NA = kidney cancer<br>NA = prostate cancer			genuinely concerned and respectful (professional caring), resulting in mutual trust and caring connection, and perceived effect as a sense of solidarity, empowerment, well-being, and healing.<br>The essential structure of an uncaring encounter emerged as: the nurse/health professional perceived as being incompetent as well as indifferent to the patient as a person and a patient, resulting in sense of mistrust and disconnection, and perceived effect as a sense of uneasiness, discouragement, and a sense of being broken down
Hargie et al. (2009) [61], Northern Ireland	1. To examine how and from what existing framing perspectives patients interpret and make sense of their initial interactions with nurses<br><br>2. To examine what discussions with cancer nurses mean to the patient	Qualitative study<br><br>Purposive<br><br>NA	Two cancer care outpatient clinics at hospitals in Norway	N = 9<br>Mean age = 59 years<br>M = 5 (55.5%)<br>N = 3 colon cancer<br>N = 2 prostatic cancer<br>N = 2 lung cancer<br>N = 1 breast cancer<br>N = 1 endocrinal organs cancer	First consultation with patients	Patients' perceptions about the role of the nurse and the overall conversation (deep-probe semi-structured "sensemaking" interviews)<br>T1 = after nurse–patient conversation	Preconceptions and conversational attitudes were frame-of-reference factors that influenced the process whereby patients refined their sense of nurse conversations.<br>In relation to preconceptions, patients could be categorised into two generic types: 'no expectations' group, who were more likely to view the nurse as a sensegiver, and 'nurse as task-related' group, who perceived the nurse

							as functional and a support person for the physician
Ivers et al. (2019) [60], Australia	1. To detect acceptability and accessibility of a cancer care service in an Australian Aboriginal health service	Qualitative study based on grounded theory<br><br>Convenience<br><br>From January 2015 to December 2016	An Australian Aboriginal community-controlled health service in New South Wales	N = 16<br>NA<br>NA<br>N = 8 Australian Aboriginal patients with cancer diagnosis/family members<br>Age: 54–81 years<br>F = 87.5%<br>NA<br>N = 8 stakeholders (N = 2 Aboriginal health workers, N = 1 registered nurse, N = 2 general practitioners, N = 2 cancer care nurses, N = 1 care coordinator)<br>NA<br>NA	Cancer care team (counsellor, health worker, and nurse, employer-funded two days per week supported by a general practitioner) supporting cancer patients from prediagnosis, at diagnosis, and through cancer treatment, coordinating with palliative care services or proving support for carers, beyond coordinating preventive activities	Acceptability of cancer service (semi-structured interviews analysed by grounded theory)*<br>T1 = during home visits<br><br>Accessibility of cancer service (semi-structured interviews analysed by grounded theory)*<br>T1 = during home visits	Improved accessibility of cancer care services, including availability of home visits, transport, and accompaniment to tertiary settings.<br>Six participants stated that the service was helpful to their well-being, relieving depression, stress, anxiety, anger, and loneliness.<br>The service was viewed as being culturally safe
Jakobsson and Holmberg (2012) [55], Sweden	1. To investigate how changing information routines might influence patients' service quality perceptions<br><br>2. To test the QPP questionnaire everyday feasibility for healthcare quality assessment	Pre/post quasi-experimental study<br><br>Consecutive<br><br>A year	Two similar wards at two hospitals in southern Sweden	N = 138<br>Mean age = 59.3 years<br>M = 94 (68.1%)<br>N = 71 (study group) newly diagnosed patients<br>Mean age = 58.9 ± 14.3<br>M = 50.1%<br>N = 67 (comparison group) newly diagnosed patients<br>Mean age = 59.7 ± 14.0<br>M = 86.0%<br>37.7% = gynaecological cancer<br>62.3%	Introduction of standardized guidelines for nursing performance and provision of oral and written information to patients (study group)<br><br>Standard care (control group)	Patients' service quality perception (QPP, a validated self-report questionnaire)<br>T0 = at diagnosis, T1 = + 6 months, T2 = + 12 months	A highest balance rating was having a private talk with nurses that increased over the 12 months ($p < 0.008$), although nurse interest in patients' worries diminished ($p < 0.004$).<br>Patients reported that they had less opportunity to take part in clinical decisions at the end than at beginning, both in medical and nursing care ($p < 0.017$).<br>The QPP indexes may be used as fairly accurate material easily

				= haematological cancer			transformed into feedback comprehensible to healthcare professionals
Jernigan et al. (2020) [34], USA	1. To explore the feasibility of a nurse-driven telephone triage intervention to improve the symptom experience of patients with cancer receiving ambulatory treatment<br><br>2. To explore trends in patients' symptom experiences	Quasi-experimental study<br><br>NA<br><br>From September 2017 to August 2018	Three ambulatory centres (breast, head and neck, and sarcoma) receiving active treatment at the University of Texas MD Anderson Cancer Centre in Houston	N = 90<br>Mean age = 52 years<br>NA<br>N = 30 breast cancer<br>N = 30 head and neck cancer<br>N = 30 sarcoma	Nurse-driven telephone triage intervention for patients receiving first-line chemotherapy	Feasibility (completion of 70% of triage calls)<br>T1 = + 1 week<br><br>Completion (compilation of MDASI and Press Ganey survey)<br>T1 = + 1 week<br><br>Symptom experience (MDASI, validated self-report questionnaire, for breast and sarcoma patients; MDASI-NH for head and neck cancer patients)<br>T0 = prior starting chemotherapy, T1 = + 1 week<br><br>Patients' satisfaction (7 questions from the PGO, not validated self-report questionnaire)<br>T1 = at midpoint of intervention, T2 = +1 week post intervention<br><br>Frequency of emergency department visits and hospital admissions for symptom management	Overall call completion rate was 78.0% (94.0% for head and neck cancer patients).<br>MDASI Interference (p = 0.002) and symptom severity ($p < 0.001$) scores were significantly different among patients in the three centres and gradually decreased over time.<br>Most patients were satisfied with the care provided by the healthcare team and did not visit the emergency department and were not admitted to hospital
Keeley et al. (2015) [89], USA	1. To determine the difference in patient satisfaction with overall nursing care and perceived nurse caring after the implementation of a	Pre-post quasi-experimental study and post-test only study	Seven inpatient units in a nursing department at a National Cancer Institute—comprehensive cancer centre	N = 280 (pre-post study design)<br>Mean Age = NA<br>Sex = NA<br>Diagnosis = NA<br>N = 158 (post-test only design)	Implementation of a nursing staff standard of care protocol including caring activities	Patient satisfaction with nursing care (HCAHPS, self-report questionnaire)<br>T0 = before implementation	Patient satisfaction with nursing care increased for some items, the most was for 'staff took my preferences and those of my family or caregiver into account in deciding

	nursing staff standard of care protocol	Probabilistic and convenience<br><br>From April 2013 to March 2014		Mean age = 60.3 ± 12.7<br>NA<br>NA		T1 = Following discharge<br><br>Perceived nursing care (CBI-24, validated self-report questionnaire)<br>T0 = before implementation<br>T1 = Before discharge	what my healthcare needs would be when I left'. Perceived nursing caring was ranked highly immediately before discharge, in particular 'encouraging the patients to call if there are problems' was the highest ranked item
Kimman et al. (2010) [90], the Netherlands	1. To investigate the cost-effectiveness of a nurse-led telephone follow-up<br><br>2. To compare patient satisfaction between nurse-led telephone and hospital follow-up	RCT, with a 2 x 2 factorial design<br><br>Consecutive<br><br>From 2005 to 2008	Seven hospitals and two radiotherapy clinics in the South of the Netherlands	N = 299<br>Mean age = 55.5 years<br>F = 299 (100.0%)<br>N = 150 (study group)<br>Mean age = 55.0 ± 9.0<br>F = 150 (100.0%)<br>N = 149 (control group)<br>Mean age = 56 ± 10.7<br>F = 149 (100.0%)<br>Breast cancer in follow-up	Nurse-led telephone follow-up (study group) + hospital visit and mammography or + educational group program<br><br>Hospital follow-up (control group) + mammography or + educational group program	Patient satisfaction (PSQ III, validated questionnaire)<br>T0, T1 = + 3 months, T2 = + 6 months, T3 = + 12 months after treatment	Nurse-led telephone follow-up had no statistically significant influence on general patient satisfaction (p = 0.379), satisfaction with technical competence (p = 0.249), or satisfaction with interpersonal aspects (p = 0.662). Regarding access of care, patient satisfaction scores were significantly higher for patients receiving telephone follow-up (p = 0.015). However, a mean difference at 12 months of 3.1 points was judged to be not clinically relevant
Kleeberg et al. (2008) [91], Germany	1. To examine how outpatient cancer patients assess their cancer care in private oncology practices and day hospitals<br><br>2. To identify the extent to which staff meet the expectations of their patients	Cross-sectional study<br><br>Consecutive<br><br>From October 2004 to January 2005	Private practices (n = 41) and day hospitals (n = 8) in Germany	N = 4,615<br>Mean age = 63.5 years<br>F = 2,630 (57.0%)<br>25% breast cancer<br>21% colorectal cancer<br>12% haematological malignancies<br>11% lymphoma	-	Patient satisfaction (PASQOC, validated self-report questionnaire)*<br>T1 = NA	The best results were obtained for the dimensions 'further support in daily life', 'nurses', and 'physician-patient-relationship'. Regarding nurses' performance, friendliness, answers to questions, and confidence have been considered as highly

							satisfying. Potential for improvement was most pronounced for 'handling of side effects', 'partnership and shared decision making', and 'communication with other patients'
Kousoulou et al. (2019) [92], Cyprus	1. To assess the levels of and explore any correlations between individualized nursing care and the quality of oncology nursing care in patients diagnosed with cancer	Descriptive correlational study<br><br>Consecutive<br><br>2017	Three different urban hospitals of Cyprus providing in-patient cancer care	N = 150<br>51–60 years = 31.0%<br>M = 85 (57.0%)<br>17% lung cancer<br>13% breast cancer<br>70% other types of cancers	Individualized nursing care	Quality of oncology nursing care (ICS, self-report validated questionnaire, and QONCS, self-report validated questionnaire)<br>T1 = NA	Data showed a medium level of support for 'Patients' individuality was provided for by nurses' (ICS-A mean = 3.41) and a high level of realization of perceived individuality in the provided care. Quality of oncology nursing care was found high on three dimensions of care ('Being supported and confirmed', 'Being respected' and 'Having a sense of belonging'). A statistically significant positive correlation was observed between the two scales of ICS (r = 0.80) and four of the dimensions of QONCS ('Being supported and confirmed', 'Being cared for religiously and spiritually', 'Sense of Belonging', and 'Being respected')
Krishnasamy (1996) [63], UK	1 To identify nursing behaviour patterns perceived of as being helpful and unhelpful by	Exploratory descriptive study<br><br>Propositive	A haematology-bone marrow transplant unit	N = 8<br>Mean age = NA<br>M = 4 (50.0%)<br>Haematological cancer	-	Supportive and unsupportive nursing behaviour (semi-structured interview analysed by thematic analysis)	Be emotionally supportive (understanding, unconditional availability, respect, intimacy, and

	hospitalized cancer patients	Two weeks				T1 = NA	companionship) and informationally support behaviour (about specific sources of stress and clarification of advice or information) have been reported as the most supportive nursing behaviours. The most frequent unsupportive behaviour was being devoid of an emotional component (conveying a negative attribution, a lack of intimacy and of respect)
Kvåle and Symnes (2013) [51], Norway	1. To reflect on whether and how health personnel, by giving good care, can function as vital resources at cancer patients' disposal in activating their general resistance resources	Interpretative qualitative study based on Antonovsky's 'Sense of Coherence' theory<br><br>Propositive<br><br>NA	An oncology ward in a regional hospital in Norway	N = 20<br>Mean age = NA<br>M = 10 (50.0%)<br>NA	-	Patients' reflections on good nursing care (not highly structured interview using an hermeneutical approach)*<br>T1 = During hospitalization	Most of patients succeeded in activating their general resistance resources in dealing with the stressor. Nurses, doctors, family, and friends can be seen to function as vital resources at their disposal when needed. Most likely good caring supported patients' promotion and maintenance of the components of meaningfulness (listening to the patients' stories about what still gives them meaning in life), comprehensibility (giving good information), and manageability (alleviation of physical suffering)

Larsson et al. (1998) [57], Sweden	1. To research associations between patient and staff dyads' ratings of importance of caring behaviours and levels of anxiety and depression	Correlational descriptive study<br><br>Consecutive<br><br>NA	Three wards at Akademiska Hospital in Uppsala	N = 106<br>Mean age = 45 years<br>F = 81 (76.4%)<br>N = 53 cancer patients<br>Mean age = 55 years<br>F = 56.6%<br>NA = neuroendocrinological cancer<br>NA = haematological cancer and lymphomas<br>NA = breast cancer<br>NA = lung cancer<br>N = 53 nurses<br>Mean age = 35 years<br>F = 96.2%	-	Patients and staff perception of importance of caring behaviour (CARE-Q, validated self-report questionnaire)<br>T1 = During hospitalization<br><br>Patient anxiety and depression (HADS, validated self-report questionnaire)<br>T1 = During hospitalization	Both groups perceived anticipatory and comforting behaviours to be among the three most important. Patients considered staff explanation and facilitation as well as anticipation to be more important than did staff. Staff rated accessibility and comforting as more important than did patients. Patient and staff ratings of the importance of staff accessibility were negatively correlated. Neither patient nor staff ratings of the importance of caring behaviours were associated with the ratings of anxiety or depression of specific patients<br>The ratings of caring behaviours were not associated with the ratings of patient anxiety or depression, neither in the patient nor staff group
MacLeod et al. (2007) [58], UK	1. To assess clinical effectiveness of a nurse-/pharmacy-led clinic for the provision of home-based oral capecitabine<br><br>2. To assess experiences of the	Prospective audit and retrospective survey**<br><br>Consecutive<br><br>From March 2003 to June 2004	A nurse-/pharmacy-led clinic at the Beatson Oncology Centre	N = 52 with metastatic colorectal cancer<br>Median age = 68 years<br>M = 33 (63.0%)<br>N = 35 colon cancer<br>N = 12 rectum cancer<br>N = 5 unknown	Nurse-/pharmacy-led clinic for the provision of home-based oral capecitabine	Clinical effectiveness of the nurse-led clinic (capecitabine dosing schedule, number of treatment modifications, response to treatment, occurrence of adverse events, and need for consultations with general practitioners)	79% of patients completed at least three cycles of treatment, 27% at least six cycles. Capecitabine dose was reduced on at least one occasion in 29% of patients and 30% experienced at least one delay. 25% of patients

	clinic from patients' perspectives			**N = 42 participated in the survey		T1 = after at least three cycles of capecitabine<br><br>Patients' experiences of clinic (non-validated questionnaire)<br>T1 = 1–4 weeks after capecitabine treatment completion	called the contact telephone numbers to ask for advice about treatment-related adverse events. 19% of patients attended hospital during the treatment and 23% had consultations with a general practitioner. Patient satisfaction of nurse-led clinic was high: 85% thought that the service provision was useful and well organised, 96% that written information provided was useful, and 85% that care provided by nurse/pharmacist was satisfying
Mårtensson et al. (2010) [69], Sweden	1. To investigate whether patient–nurse dis/agreement concerning cancer patients' situations was of importance to patients' satisfaction with care<br><br>2. To describe cancer patients' satisfaction with care and investigate its relationship to cancer patients' emotional distress	Prospective study<br><br>Consecutive<br><br>Three days per patient–nurse dyad from January 2005 to December 2005	Three oncological wards and two haematological wards in two hospitals in Sweden	N = 82 dyads of cancer patients newly admitted to ward with a nurse responsible for their care (N = 142)<br>Mean age = 52.3 years<br>F = 86 (60.5%)<br>N = 90 patients<br>Mean age = 60 years<br>M = 60.0%<br>44.0% haematological neoplasms<br>9.0% hepar/renis cancer<br>9.0% sarcoma<br>5.0% prostate/testis cancer<br>5.0 % lung cancer<br>5.0% gastrointestinal cancer	Nursing care delivered for three days	Cancer patients' satisfaction with received care from the specific nurse (NSC, validated self-report questionnaire)<br>T1 = on the 3rd day<br><br>Cancer patients' satisfaction with received medical and nursing care and the service (CASC, validated self-report questionnaire, and two open questions)<br>T1 = on the 3rd day<br><br>Emotional distress (HADS, self-report validated questionnaire)	The patients cared for by a nurse who underestimated their level of depression were significantly less satisfied with the care they received from that specific nurse than patients cared for by a nurse who was in agreement with or overestimated patients' levels of depression. No differences in NSC emerged regarding patient–nurse dis/agreement concerning patients' anxiety, coping resources, or QOL.

				N = 52 nurses<br>Mean age = 39 years<br>F = 96.1%		T0 = after admission<br><br>Coping resources (CBI, self-report validated questionnaire)<br>T0 = after admission<br><br>QOL (FACIT-Sp, validated self-report questionnaire)<br>T0 = after admission	There were no differences between the patient subgroups' consistent agreement, consistent disagreement, and mixed agreement with regard to their satisfaction with care received from a specific nurse (NSC), from the nurses as a group (CASC), or their general satisfaction with care. The known phenomenon of nurse overestimation of cancer patients' problems did not appear to be of importance to patients' satisfaction with care. Anxious and depressed patients were less satisfied with some aspects of the care than the remaining patients
Martinez et al. (2015) [83], USA	1. To examine differences in healthcare service utilization among patients with advanced cancer participating in a nurse-led psychoeducational intervention	Secondary analysis of a RCT<br><br>Consecutive<br><br>3 months along 2013	Four Michigan cancer centres, at home	N = 484 with advanced cancer<br>Mean age = 60.5 years<br>F = 300 (62.0%)<br>N = 159 (34.0%, first study group)<br>N = 162 (33.0%, second study group)<br>N = 163, 34.0%, control group)<br>33.0% = breast cancer<br>29.0% = lung cancer<br>25.0% = colorectal cancer<br>13.0% = prostate cancer	Brief nurse-led psychoeducational intervention to improve coping (first study group)<br><br>Extensive nurse-led psychoeducational intervention to improve coping (second study group)<br><br>Usual care (control group)	Patients' healthcare service utilization (emergency department visits or inpatient hospitalizations)<br>T0, T1 = +3 months, T2 = +6 months after treatment	No significant differences in emergency department visits or inpatient hospitalizations were observed among the study arms, nor in the adjusted analysis

McCorkle et al. (2000) [93], USA	1. To compare the length of survival of older post-surgical cancer patients who received a specialized home care intervention provided by advanced practice nurses with that of patients who received usual follow-up care in an ambulatory setting<br><br>2. To assess potential predictors of survival in terms of depressive symptoms, symptom distress, functional status, comorbidities, length of hospital stay, age of patient, and stage of disease	RCT<br><br>Consecutive<br><br>From February 1993 to December 1995	A comprehensive Cancer Centre in south-eastern Pennsylvania	N = 375 older post-surgical solid cancer patients<br>Mean age = NA<br>F = 195 (52.0%)<br>N = 190 (study group)<br>65 years or older = 63.2%<br>M = 51.6%<br>N = 185 (control group)<br>65 years or older = 68.1%<br>F = 55.7%<br>30.5% = breast cancer<br>38.9% = lung cancer<br>23.5% = colorectal cancer<br>37.3% = prostate cancer<br>17.0% = head/neck cancer<br>52.3% = others	Home care intervention by advanced nursing practice consisted of three home visits and five telephone contacts (study group)<br><br>Usual follow-up care in ambulatory setting (control group)	Survival (time in days from enrolment into the study until death or last date known alive)<br>T0, T1 = +3 months, T2 = +6 months after hospitalization<br><br>Depressive symptoms (CES-D, self-report questionnaire)<br>T0, T1 = +3 months, T2 = +6 months after hospitalization<br><br>Symptom distress (SDS, self-report questionnaire)<br>T0, T1 = +3 months, T2 = +6 months after hospitalization<br><br>Functional status (ESDS, self-report questionnaire)<br>T0, T1 = +3 months, T2 = +6 months after hospitalization	24.8% of patients died: 22% of those who died were patients in the specialized home care intervention group, compared with 28% in the usual care group. The specialized home care intervention group was found to have increased survival (p = 0.002) among late-stage patients (2-year survival was 66.7% compared with 39.6%, $p < 0.05$), while among early stage patients only, there was no difference in survival between the groups. Adjusting for significant baseline covariates, the relative hazard of death in the usual care group was 2.04 ($p = 0.001$). Age, race, depressive symptoms, symptom distress, and enforced social dependency at baseline were not predictive of the length of survival for this sample ($p > 0.20$)
McCorkle et al. (2015) [94], USA	1. To evaluate the effects of a multidisciplinary coordinated intervention by advanced practice nurses at the clinic level on outcomes with patients newly diagnosed with late-stage cancer	Cluster RCT<br><br>Cluster random<br><br>From August 2010 to December 2012	Four disease-specific multidisciplinary clinics; gynaecological, lung, head and neck, and gastrointestinal clinics	N = 146 newly diagnosed late-stage cancer within 100 days<br>Mean age = 60 years<br>M = 82 (56.2%)<br>NA = gynaecological cancer<br>NA = lung cancer<br>NA = head and neck cancer	10-week standardized advance nurse practice-coordinated multidisciplinary intervention (study group)<br><br>Usual care (control group)	Symptoms (SDS, validated self-report questionnaire)*<br>T0 = within first 100 days from diagnosis, T1 = + 1 month, T2 = + 3 months<br><br>Health distress (four-item scale developed by the Stanford Patient	No differences between the two groups on the primary patient-reported outcomes were observed, however, physical and emotional symptoms remained stable or significantly improved from baseline for both groups. Overall, secondary outcomes

		NA = gastrointestinal cancer		Education Research Center)*<br>T0 = within first 100 days from diagnosis, T1 = + 1 month, T2 = + 3 months<br><br>Depression (PHQ-9, validated questionnaire)*<br>T0 = within first 100 days from diagnosis, T1 = + 1 month, T2 = + 3 months<br><br>Functional status (ESDS, self-report questionnaire)*<br>T0 = within first 100 days from diagnosis, T1 = + 1 month, T2 = + 3 months<br><br>Self-rated health (SF-12, validated questionnaire)<br>T0 = within first 100 days from diagnosis, T1 = + 1 month, T2 = + 3 months<br><br>QOL (FACT-G, validated questionnaire)<br>T1 = + 1 month, T2 = + 3 months<br><br>Anxiety (HADS, validated self-report questionnaire)<br>T1 = + 1 month, T2 = + 3 months<br><br>Uncertainty (MUIS-C, validated questionnaire)	remained stable within the groups. However, patients in the control group reported significantly better self-efficacy at one month ($p < 0.0097$) and less uncertainty at one month ($p < 0.0007$) and three months ($p < 0.0106$) compared to those in the study group

						T1 = + 1 month, T2 = + 3 months<br><br>Self-efficacy (SEMCD 6, validated questionnaire)<br>T1 = + 1 month, T2 = + 3 months	
McGrath (2000) [95],<br>Australia	1. To record the participants' understanding of the positive and negative aspects and outcomes of course attendance	Prospective study<br><br>Consecutive<br><br>First half of 1999	Leukaemia Foundation of Queensland	N = 23 patients with haematological malignancies and their families<br>N = 15 (57.7%) patients<br>N = 8 (30.7%) relatives<br>40–49 years = 23.1%<br>F = 19 (73.1%)<br>NA	Multidisciplinary educational support course, combining education, discussion, practical exercises, and social support (Taking Control)	Personal experience of the course (author-designated, self-report questionnaire with Likert scales and open-ended comments)*	Participants experience a high level of satisfaction, gain substantially in terms of knowledge, coping skills, and a positive framework within which to view their illness.<br>Many obtain great value from the opportunity to meet and talk with others in similar situations
Milani et al. (2013) [96],<br>Italy	1. To test the feasibility of an ONMDS-based on NSOs<br><br>2. To integrate the ONMDS in a nursing record implementing nursing plan and to tailor nursing care to the collected data	Pre/post- test study<br><br>NA<br><br>From May 2010 to November 2012	Medical area of European Institute of Oncology in Milan	N = 50 medical records from medical oncological patients<br>NA<br>NA<br>Oncological and haematological malignancy	Development and implementation of a new nursing record based on ONMDS	Care of patients (standard JCI: nursing care is planned within 24 h from admittance, nursing care is tailored using the collected data, nursing plan is updated and modified on the basis of patient reassessment)<br>T0, T1 = + 3 months from ONMDS introduction<br><br>Assessment of patients (standard JCI: patients' needs are identified on the basis of nursing and medical assessment, and they are registered; all patients underwent a screening of pain; the patient is subjected to	The ONMDS is composed of 49 NSOs clustered into 15 categories (gastrointestinal outcomes, genitourinary outcomes, respiratory outcomes, skin outcomes, fluid and electrolyte balance outcomes, neurological outcomes, security, functional status, vascular access outcomes, nutritional status, pain, psychosocial discomfort, activities of daily living, instrumental activities daily living, and self-care outcomes).

						revaluation in order to determine the response to treatment; the patient is subjected to revaluation in order to plan for continuity of care; the patient is subjected to revaluation at appropriate intervals depending on the treatment plan and identified needs)<br>T0, T1 = + 3 months from ONMDS introduction	The introduction of the ONMDS in clinical practice showed a significant improvement of the JCI standards in nursing records (*p* < 0.0001)
Molassiotis et al. (2021) [22], China	1. To explore the effectiveness of nurse-led advanced practice for patients with cancer	Scoping review of RCTs or cluster trials<br><br>-<br><br>Studies from 2001 to 2019	Oncology nurse-led clinics and advanced practice in outpatient settings	N = 17 studies<br>NA<br>NA<br>NA	Nurse-led advanced practice clinics	Patients' symptoms (FACT-HNSI, CSAS, NRS and BFI-I for fatigue)<br>NA<br><br>Distress levels (HADS, POMS, CES-D, STAI)<br>NA<br><br>Satisfaction (satisfaction scale, VAS)<br>NA<br><br>QOL (EORTC-QLQ-C30, FACT-HN, FACT-G, FACT-B/ES, QLQ-BR23)<br>NA	The results support the effectiveness of nurse-led clinics in improving self-reported responses such as distress levels, satisfaction, quality of life, depressive symptoms, concerns, and vomiting among cancer patients. Patients are consistently highly satisfied with nurse-led clinic care and their information needs are more fully met with nurse-led care as well as there is more shared decision-making
Møller et al. (2005) [97], Denmark	1. To investigate the impact of patient education regarding provision of their own catheter care on the frequency of CRIs	RCT<br><br>Consecutive<br><br>From May 2002 to September 2002	A specialized haematological unit at the University Hospital of Copenhagen	N = 82 with tunnelled double-lumen Hickman catheters with haematological malignancies<br>Mean age = NA<br>M = 49 (59.7%)<br>N = 42 (study group)<br>Median age = 51 years<br>M = 25 (59.5%)	Step-by-step individualized training and supervision by a clinical nurse specialist to become independent in CVC care (study group)<br><br>Standard CVC procedures carried out by nurses inside and	Incidence of CRIs (clinical criteria and laboratory data)<br>T1 = at clinical presentation of CRIs	A significant reduction in CRIs was found in the study group, with a > 50% reduction in the incidence rate of CRIs (*p* < 0.01)

				N = 40 (control group)<br>Median age = 49<br>M = 24 (60.0%)<br>N = 41 acute myeloid leukaemia<br>N = 16 lymphoma<br>N = 11 acute lymphoid leukaemia<br>N = 6 myelodysplastic syndrome<br>N = 2 myeloproliferative disease<br>N = 6 myeloma	outside the hospital (control group)		
Muñoz et al. (2018) [98], USA	1. To determine whether the inclusion of a gastrointestinal oncology nurse navigator on the multidisciplinary cancer care team is associated with improved quality of care for patients	Retrospective cohort study<br><br>Random<br><br>2014–2016	Community Medical Centres Healthcare Network in Fresno County in California	N = 120<br>Mean age = 63.8 years<br>M = 69 (57.5%)<br>N = 60 (study group)<br>Mean age = 60.6 years<br>M = 34 (56.7%)<br>N = 60 (control group)<br>Mean age = 67.1 years<br>M = 35 (58.3%)<br>100.0% = gastrointestinal cancer	Inclusion of a gastrointestinal oncology nurse navigator on the multidisciplinary cancer care model (study group)<br><br>Multidisciplinary cancer care model (control group)	Quality of care model (time from diagnosis to treatment through Epic electronic health records system and average number of missed appointments through the Epic electronic health records system)<br>NA	Patients with an oncology nurse navigator had a shorter time lapse between diagnosis and treatment commencement ($p < 0.001$). In this group, the average time spent between initial diagnosis and the start of treatment was 15.15 days, compared to 42.93 days for patients who were not part of the multidisciplinary cancer care model. Statistical analysis revealed no difference in missed appointment rates between the two groups ($p = 0.7$)
Musiello et al. (2017) [26], Australia	1. To explore the prevalence of distress, type of problems experienced by haematological	Cross-sectional prospective pilot study<br><br>Consecutive	An outpatient haematological oncology clinic in Western Australia	N = 68 with haematological cancer attending outpatient treatments<br>Mean age = 52 years<br>F = 32 (53.0%)	Routine distress screening performed by either a register nurse or a clinical psychologist register	Distress (NCCN DT, self-report validated questionnaire, and the Problem List, self-report validated questionnaire)	40% reported significant distress. All patients reported physical problems and 72% reported emotional problems—the major

	patients, and referrals for supportive care<br><br>2. To explore effects of demographic and clinical variables on distress<br><br>3. To explore effect on the time of health professionals conducting the screening in the ambulatory chemotherapy setting	2011–2012		N = 52 lymphoma<br>N = 9 myeloma<br>N = 7 leukaemia		While patients received chemotherapy<br><br>Time conducting the screening in the ambulatory<br>While patients received chemotherapy	contributors to distress and to time spent with the health professional. Distress was unrelated to age, gender, or cancer type. Patients were less likely to have significant distress at the end of treatment than at the beginning (OR = 0.15). The psychologist spent less time with patients compared to the nurse (18 vs 48 min, $p < 0.001$). The more emotional problems reported, the greater the time spent with the patient ($p = 0.009$)
Naveh et al. (2011) [99], Israel	1. To examine pain severity, satisfaction with pain management, and patient-related barriers to pain management among patients with cancer in oncology units at a teaching hospital in Israel	Descriptive, cross-sectional, correlational study<br><br>Convenience<br><br>From January 2008 to December 2008	Oncology division of a large teaching hospital in Israel	N = 144 experiencing pain in the last 24 hours<br>N = 76 (ambulatory patients)<br>N = 68 (inpatients)<br>Mean age = 53.2 ± 14.9 years<br>F = 73 (50.7%)<br>N = 61 solid cancers<br>N = 35 haematological cancer<br>N = 18 bone marrow transplantation	Administration of the questionnaires by nurses or research nurses	Pain severity (RPS-POQ, validated self-report questionnaire)<br>During hospitalization/outpatie nt basis<br><br>Satisfaction with pain management (RPS-POQ, validated self-report questionnaire)<br>During hospitalization/outpatie nt basis<br><br>Patient-related barriers to pain management (validated self-report questionnaire, BQ-SF)<br>During hospitalization/outpatie nt basis	A significant inverse relationship was observed between patients' pain severity and expectation of pain relief. Less-educated patients had significantly higher pain severity scores. The greatest barriers to pain control were fear of addiction and notion that medication should be saved in case the pain gets worse.<br>A difference was observed between patients' levels of satisfaction with nurses' and doctors' treatment of their pain and the amount of time they had to wait after asking for more or different

							medication for their pain. The differences in satisfaction levels were significant for nurses (U = 124, p = 0.056) and doctors (U = 117, p = 0.026)
Oleske and Hauck (1988) [16], USA	1. To determine if the introduction of modest changes in the home health system would affect certain cancer patient patterns of care and outcomes	Pre/post-test study<br><br>Cluster random<br><br>From June 1980 to August 1983	29 Medicare-certified home health agencies in two health planning regions of Illinois	N = 874 cancer patients referred to home care<br>Mean age = 70 years<br>NA<br>22.0% = colorectal cancer<br>19.0% = lung cancer<br>11.0% = breast cancer<br>48.0% = others	Oncology nurse specialist with continuing education on cancer (study group 1)<br><br>Continuing education on cancer alone (study group 2)<br><br>Only observation (control group)	Duration of care (time in days counted from the first health nurse visit to the last home health nurse visit for an admission to agency care)<br>T1 = at the end of the study<br><br>Number of visits by the home health nurse (number of home visits made by home health nurse)<br>T1 = at the end of the study<br><br>Physiologic complications (presence of urinary tract infection, respiratory tract infection, skin/mucocutaneous infection, bleeding, febrile state, thrombophlebitis, or pulmonary embolus)<br>T1 = at the end of the study<br><br>Hospitalization<br>T1 = at the end of the study	The group that received the services of the oncology nurse specialist and continuing education on cancer demonstrated the largest percentage increase in cancer patient referrals to home care and experienced a significant decrease in cancer patient mortality. No significant difference among cancer patients in the three study groups was noted for hospitalization rate or incidence of physiologic complications while on agency caseload

						Disposition at discharge (status of the patient at the time of the nurse's last contact with the patient for the purpose of terminating home care)<br>T1 = at the end of the study<br><br>Referral rate (number of cancer patients referred to home care)<br>T1 = at the end of the study	
Panteli and Patistea (2007) [68],<br>Greece	1. To investigate the quality of pain management provided in a Hellenic inpatient oncology setting. The intention was to provide baseline data in this area because there has been no study that evaluates the quality of pain management in Greece	Descriptive, cross-sectional, correlational study<br><br>Convenience<br><br>NA	A large oncology hospital in Athens	N = 70 patients experiencing pain 24 hours prior to completion of the questionnaires<br>Mean age = 62.7 ± 16.8 years<br>F = 36 (51.4%)<br>N = 15 gynaecological/breast cancer<br>N = 15 lung cancer<br>N = 13 gastrointestinal cancer<br>N = 12 genitourinary cancer<br>N = 5 pancreas cancer<br>N = 3 sarcoma<br>N = 3 occult primary malignancy<br>N = 2 myeloma<br>N = 2 bone cancer	Cancer-related pain management	Patients' satisfaction with care (APSP OQ, validated tool)*<br>NA<br><br>Pain intensity (VAS, self-report validated tool)*<br>NA<br><br>Pain relief (VAS, self-report validated tool)*<br>NA<br><br>Quality of interpersonal relationships with nurses (qualitative comments analysed by content analysis)*<br>NA	Despite the high levels of pain observed indicating ineffective treatment, patients reported to be 'satisfied' or 'very satisfied' with the management of their pain and the general care given. Several significant differences and relationships were found between the variables examined. Nurses had developed therapeutic relationships and effective communication with the patients. Further, qualitative comments provided rich data of rational and humanistic aspects of nursing
Predeger et al. (2014) [100],<br>USA	1. To evaluate an oncology rehabilitation program over time	Cross-sectional, retrospective program evaluation	An outpatient oncology rehabilitation program housed in	N = 102 cancer survivors who completed a 10-week oncology	An oncology multidisciplinary rehabilitation program per 10 weeks	Health-related QOL (SF-36)*<br>T0, T1 = at the end of the program*	A small increase in level of activity from precancer to the current time (post-

		using a mixed-methods approach<br><br>Consecutive<br><br>2.5 years	an urban, hospital-based comprehensive cancer centre in Alaska	rehabilitation program<br>Mean age = 62.9 ± 8.91 years<br>F = 73 (76.1%)<br>N = 58 breast cancer<br>N = 13 ovarian or uterine cancer<br>N = 5 lung cancer<br>N = 5 prostate cancer		Activity level and perceived health status (28-item retrospective survey, not validated)*<br>T1 = at the end of the program<br><br>Motivators (checklist and open-ended questions)*<br>T1 = at the end of the program<br><br>Barriers (open-ended questions)*<br>T1 = at the end of the program	rehabilitation) (z = –3.1, *p* < 0.01) was reported.<br>Most and least helpful motivators emerged from the narrative data, resulting in the shared interpretation of 'I have my life back'. Survivors who complete a rehabilitation program retain a sense of restoration and caring, and some engage in physical activity over time.<br>Personal attention from both the nurse and the physical therapist was huge; patients reported that they remembered themselves week after week
Riese et al. (2017) [31],<br>Germany	1. To evaluate the impact of a standardized patient education program provided by specially trained oncology nurses on therapy management regarding side effects and unplanned therapy interruptions	RCT<br><br>Cluster consecutive<br><br>From March 2014 to October 2014	28 office-based oncology practices all over Germany	N = 165 newly adjusted on an oral anti-cancer therapy<br>Mean age = 69.9 years<br>F = 89 (53.9.%)<br>N = 111 (study group)<br>Mean age = 69.3 ± 23.3<br>F = 62 (55.9%)<br>N = 54 (control group)<br>Mean age = 71.3 ± 10.9<br>F = 27 (50.0%)<br>N = 23 colon cancer<br>N = 19 myeloproliferative neoplasms<br>N = 15 breast cancer<br>N = 12 lung cancer	Usual oncologist counselling in addition to a standardized patient education program provided by specially trained oncology nurses on therapy management (study group)<br><br>Usual oncologist counselling (control group)	Therapy-related side effects (frequencies from the patients' diary)<br>T0, T1 + 2 weeks from the first educational appointment, T2 = + 2 weeks from T1, T3 = + 3 weeks from T2, T4 =+ 3 months after from T3, at the end of the follow-up period<br><br>Unplanned therapy interruptions (frequencies from the patients' diaries and nurses' records)<br>T0, T1 + 2 weeks from the first educational appointment, T2 = + 2 weeks from T1, T3 = + 3	Patients of the study group reported fewer side effects (skin rash, pain, fatigue, nausea, vomiting), tending to handle side effects and critical situations better.<br>Patients in the standard care group interrupted the therapy more frequently without informing their oncologist, compared to the study group

						weeks from T2, T4 =+ 3 months after from T3, at the end of the follow-up period	
Scott et al. (2019) [48], USA	1. To assess the effect of an education intervention on nurses' use of the teach-back method, as well as the effects on patient satisfaction at discharge	Quasi-experimental pre-post study<br><br>Cluster convenience<br><br>NA	A 20-bed inpatient adult haematology-oncology unit in a comprehensive cancer centre within an academic medical centre in the southeastern USA	N = 19 nurses<br>47.0% had more than five year experience<br>NA<br>NA<br>NA = patients<br>adult patients receiving cancer treatment<br>NA<br>NA	Implementation of the teach-back method delivered by nurses	Nurses' understanding of the teach-back (CCS, questionnaire)<br>T0, T1 = post implementation, T2 = + 1 month post implementation, T3 = + 3 months post implementation<br><br>Patient satisfaction (PGI, validated questionnaire)<br>T0 = - 3 month before implementation, T1 = + 3 months post implementation	The results of this study indicated that nurses were more confident in their ability to use the teach-back method and integrated many teach-back competencies into clinical practice. Although few follow-up surveys were received, longer-term data indicated continued improvement in patient satisfaction and understanding of discharge instructions
Skrutkowski et al. (2008) [78], Canada	1. To examine the impact on continuity of nursing care delivered by a pivot nurse in oncology to improve symptom relief and outcomes for patients with lung or breast cancer	RCT<br><br>Consecutive<br><br>Six months	Three outpatient ambulatory oncology clinics in a large university health centre in Quebec	N = 190<br>Mean age = 59.9 years<br>F = 131 (68.9%)<br>N = 93 with lung cancer (study group)<br>Mean age = 60.5 ± 11.1 years<br>F = 66 (71.0%)<br>N = 97 with breast cancer (control group)<br>Mean age = 59.3 ± 10.7 years<br>F = 65 (67.0%)	Nursing care delivered by a pivot nurse in oncology (study group)<br><br>Usual care (Control group)	Symptom distress (SDS, validated self-report questionnaire)<br>Every three weeks<br><br>Fatigue (BFI, self-report validated questionnaire)<br>Every three weeks<br><br>QOL (FACT-G 4, validated self-report questionnaire)<br>Every three weeks<br><br>Use of healthcare resources (clinical appointments, emergency departments visits, hospitalizations from the hospital records)<br>Every three weeks	No significant differences in symptom distress, fatigue, quality of life, and healthcare usage exists between groups. The new nursing role did not have an impact on the patient outcomes under study

Sommer et al. (2021) [62], Denmark	1. To explore haematological cancer survivors' experiences of participating in a shared care follow-up based on alternating routine physician visits and nurse-led telephone consultations	Exploratory qualitative interview study<br><br>Purposeful<br><br>From January 2019 to April 2019	The Department of Haematology of Aalborg University Hospital in Denmark	N = 12 with B cell neoplasms in remission or stable without treatment interventions for at least 6 months<br>Mean age = 64 years<br>F = 6 (50.0%)<br>N = 5 follicular lymphoma<br>N = 4 marginal zona lymphoma<br>N = 2 diffuse large B cell lymphoma<br>N = 1 chronic lymphocytic leukaemia	Shared care follow-up based on alternating routine physician visits and nurse-led telephone consultations	Patients' perceptions and experiences of participation in shared care follow-up (semi-structured interviews analysed by thematic analysis)*<br>During the study period	Findings suggest that haematological patients found nurse-led telephone consultations convenient and helped them to alleviate anxiety. Despite fewer visits to the hospital and fewer physical examinations, the patients' sense of security was maintained. Completing questionnaires and emotional and psychosocial focus in nurse consultations were considered beneficial; moreover, telephone use was considered as personal and an acceptable way of talking about topics of a sensitive nature
Stacey et al. (2021) [49], Canada	1. To evaluate cancer symptom management associated with telephone-based nursing services, in particular the quality of symptom support provided by nurses for patients with and without the use of COSTaRS practice guides and the measure of the impact of nursing telephone-based practice on patient	Quality improvement project<br><br>Convenience<br><br>From January 2018 to February 2018	A large Canadian ambulatory oncology program embedded within an academic teaching hospital	N = 62 patients with cancer treatment-related symptoms (113 audio-records calls, of whom 63 with marched documentation)<br>Mean age = 64 years<br>F = 40 (60.6%)<br>N = 25 breast cancer<br>N = 13 gastrointestinal cancer<br>N = 7 lung cancer<br>N = 18 others	Telephone-based oncology nursing services based on COSTaRS guidelines	Quality of cancer symptom management (SMAT, an 8-item tool to analyse audio-recorded calls and documentation)<br>From 6 weeks before the calls to 6 weeks after the calls<br><br>Patients' outcomes (SMAT)<br>From 6 weeks before the calls to 6 weeks after the calls	Of 113 audio-recorded calls, 58.0% had COSTaRS symptoms. Of them, 95% were documented. Average SMAT quality score was 71% for audio-recordings and 63% for documentation of calls. COSTaRS practice guide use was documented in 33% of calls. Patient outcomes indicated symptoms were resolved (38%), worse (25%), unchanged (3%), or unknown (33%).

	and health system outcomes					Healthcare system use (emergency department visits)<br>From 6 weeks before the calls to 6 weeks after the calls	13% of patients had an emergency department visit within 14-days post that was related to the symptom discussed
Stuart et al. (2019) [101],<br>USA	1. To examine the impact of three teaching strategies on patients' knowledge and understanding of their diagnosis	Prospective pre-/post-test pilot study<br><br>Consecutive<br><br>Seven months	A clinic in Texas	N = 30 patients with brain neoplasms who had an existing neuro-oncology treatment plan at the enrolling clinic<br>Mean age = 48.8 ± 16.2 years<br>F = 16 (53.3%)<br>N = 10 (strategy 1)<br>NA<br>NA<br>N = 10 (strategy 2)<br>NA<br>NA<br>N = 10 (strategy 3)<br>NA<br>NA<br>60% = glioblastoma<br>16.7% = astrocytoma<br>10% = glioma<br>13.3% = other	Patient education/information about the tools by a nurse (strategy 1 = standard of care)<br><br>Patient education/information about the tools by a nurse (strategy 2 = standard of care + educational binder)<br><br>Patient education/ information about the tools (strategy 3 = standard of care + educational binder + Cancergraph)	Patients' knowledge and confidence in understanding their diagnosis (developed survey)<br>T0, T1 = + 2 weeks after initial intervention<br><br>Participants' amount of contact (follow-up; chart review of participants' records)<br>During the study period	Patients who received strategy 3 had higher mean post-test knowledge scores compared to those who received strategy 1 or 2, but the difference was not statistically significant ($p = 0.16$).<br>In addition, fewer calls were made to the healthcare team in the strategy 3 group compared to the strategy 1 and 2 groups, but this was not statistically significant ($p = 0.14$)
Tseng and Moorhead (2014) [102],<br>USA	1. To identify nursing interventions that provide safety for oncology care by exploring recognized nursing intervention in the format of standardized terminology in electronic health records	Secondary analysis, descriptive retrospective study<br><br>Convenience<br><br>From June to December 2010	4 oncology units in a tertiary hospital in Iowa	N = 2,237 cancer patients with planned nursing care documented and related to risk factor<br>Mean age = 55 ± 17 years<br>F = 1,410 (63.0%)<br>NA	Nursing interventions relevant to Safety (as defined by NIC2)	Nursing interventions in the domain of Safety (observation and analysis of patients' using Electronic health records)<br>NA<br><br>Fall prevention: behaviour (NOC3)<br>At admission and discharge<br><br>Infection severity (NOC3)	11 nursing interventions in the domain of safety of NIC related to two classes: Risk Management and Crisis Management.<br>Fall prevention is the most frequently found intervention, followed by Infection Protection and Infection Control.<br>Patients receiving Fall Prevention as nursing interventions show older and longer length

						At admission and discharge<br><br>Tissue integrity: skin and mucous membranes (NOC3)<br>At admission and discharge	of stay than the overall sample. For the outcomes 'Fall Prevention: Behavior' and 'Knowledge: Fall Prevention' related to 'Fall Prevention' intervention, the rating at admission (569) is close to the rating at discharge (322), which mean 247 patients do not receive following evaluation for rating outcome. Thus, not every patient receiving an intervention has been rated for outcome. Moreover, the rate of follow-up evaluation is largely dropping
Westman et al. (2019) [103], Sweden	1. To compare patients' perception of received health-related information, access to supportive care resources, patient involvement and care coordination before and after introduction of a new advanced nursing role in cancer care	Cross-sectional study with two cohorts<br><br>Convenience<br><br>From April 2015 to April 2017	In the Stockholm Gotland area, Sweden region of Sweden	N = 1,872<br>Mean age = NA<br>F = 1,068 (57.0%)<br>N = 869 (baseline data)<br>≥65 years: 69%<br>F = 500 (58.0%)<br>N = 1,003 (follow-up data)<br>≥65 years: 63%<br>F = 568 (60.0%)<br>N = 32% gynaecological cancer<br>N = 25% haematological cancer<br>N = 22% upper gastrointestinal cancer<br>N = 21% head and neck cancer	Standard care (baseline data)<br><br>Introduction of the coordination contact nurse role (follow-up data)	Patients' perception of health -related information (EORTC QLQ-INFO25, validated questionnaire)<br>T2, T1 = at follow-up<br><br>Access to supportive care resources (7 items of the study specific questionnaire)<br>T2, T1 = at follow-up<br><br>Patients' involvement in their own care (5 items of the study specific questionnaire)<br>T2, T1 = at follow-up<br><br>Patients' experience of coordination (6 items of	Results show statistically significant patient-reported improvements after the introduction of the coordination contact nurse role, regarding health-related patient information ($p = 0.0006$). Statistically significant improvements were even seen related to availability of supportive care resources ($p \leq 0.0001$) and individual written care plans ($p < 0.0001$). Regarding coordination of care, involvement in care transitions declined in the follow-up data ($p = 0.01$), while the

						the study specific questionnaire)<br>T2, T1 = at follow-up	patients' view of how the health care providers transferred information between acute care and palliative care improved (p = 0.011)
Williams (1998) [18],<br>USA	1. To examine the dimensions of nurse caring that contribute to patients' perceptions of quality and nursing caring behaviours	Narrative review<br><br>NA<br><br>NA	An inpatient medical unit and an outpatient oncology clinic connected with a large south-eastern regional medical center in North Carolina	N = 259 (3 studies)<br>Mean age = 52.7 years<br>F = 164 (63.3%)<br>N = 94 medical cancer inpatients (first study)<br>Mean age = 50.5 ± 14.5 years<br>F = 49 (52.1%)<br>NA<br>N = 77 oncology outpatients (second study)<br>Mean age = 52.9 ± 14.4 years<br>F = 56 (72.7%)<br>NA<br>N = 88 oncology patients (third study)<br>Mean age = 55 years<br>F = 59 (67.0%)<br>NA	Holistic nursing care	Humanistic caring component of the health care provider-patient interaction (HCI, validated questionnaire)<br>NA	Patients perceived less spiritual and interpretive caring and more physical and sensitive caring from nurses.<br>Patients were able to identify caring behaviours and attitudes from nurses and placed more emphasis on care that recognized them as unique individuals with a need to share feelings, to have someone listen to them and to be accepting them
Yates et al. (2004) [24],<br>Australia	1. To evaluate the effectiveness of an educational intervention in overcoming attitudinal barriers and improving ambulatory cancer patients' ability to more effectively prevent and manage pain	RCT<br><br>Consecutive<br><br>From May 1999 to November 2000	Ambulatory oncology clinics at two tertiary hospitals	N = 189<br>NA<br>NA<br>N = 97 (study group) ambulatory cancer patients with cancer related pain<br>45–59 years = 52.6%<br>NA<br>N = 92 (control group) ambulatory cancer patients with cancer related pain<br>45–59 years = 44.6%	Two-session PMI, instructional and cognitive behavioural strategies with information about pain and pain management by two experienced nurses (treatment group)<br><br>General patient education intervention (control group)	Patients' attitudes and beliefs that may influence pain responses (self-report questionnaire developed for the study)<br>T0 (prior to randomization), T1 = + 1 week post intervention, T2 = + 2 months post intervention<br><br>Patients' knowledge about medications and	One-week post-intervention, patients receiving the PMI had a significantly greater increase in self-reported pain knowledge, number of pain treatments recommended, perceived control over pain, reduction in concerns about addiction, side effects, developing tolerance to

		NA<br>43.9 % breast cancer<br>24.3% colorectal cancer<br>17.5% lung cancer<br>14.3% head and neck cancer		side effects (two items of the study questionnaire)<br>T0, T1 = + 1 week post intervention, T2 = + 2 months post intervention<br><br>Patients' perceived self-efficacy (two subscales of the study questionnaire)<br>T0, T1 = + 1 week post intervention, T2 = + 2 months post intervention<br><br>Patients' pain experiences (BPI, validated questionnaire)<br>T0, T1 = + 1 week post intervention, T2 = + 2 months post intervention<br><br>Patients' wellbeing (HADS, validated questionnaire; EORTC QLQ-30, validated questionnaire)<br>T0, T1 = + 1 week post intervention, T2 = + 2 months post intervention<br><br>Patients' satisfaction (PSQ, validated questionnaire)<br>T0, T1 = + 1 week post intervention, T2 = + 2 months post intervention	pain relieving medication, and willingness to tolerate pain.<br>No significant differences were noted on secondary outcome measures. From baseline to T2, patients who received the intervention continued to report greater reduction in concerns about addiction, tolerance, and willingness to tolerate pain. In addition, they also reported a greater reduction in levels of anxiety and fatalistic views about their pain. However, change scores for the variables assessing feelings of control were not significantly different between groups

Zou et al. (2016) [66], China	1. To determine whether sweet potato can alleviate constipation in leukaemia patients undergoing chemotherapy	RCT<br><br>Consecutive<br><br>From October 2012 to June 2013	Department of Hematology of the First Affiliated Hospital of Soochow University	N = 120<br>Mean age = 46.7 years<br>M = 66 (55.0%)<br>N = 57 (study group)<br>Mean age = 47.4 ± 12.0<br>M = 56.1%<br>N = 63 (control group)<br>Mean age = 46.1 ± 11.4<br>M = 54.0%<br>Acute myeloid and lymphocytic leukaemia	Routine nursing method (abdominal massage, increased water intake, appropriate physical activities, dietary modification, laxatives or enemas if patients showed symptoms of constipation) plus boiled or congee sweet potato *Ipomea batatas* consumption (100 gr/twice a day from admission to discharge, in the study group) from the 1st day following admission to discharge<br><br>Routine nursing methods (the same, in the control group)	Constipation and defecation (Roma III validated criteria)<br>T0 = on admission, T1 = + 2 days, T2 = +5 days after chemotherapy initiation, T3 = at discharge<br><br>Satisfaction with bowel movements (using a 3-degree score)<br>T1 = + 2 days, T2 = +5 days after chemotherapy initiation, T3 = at discharge	On the 2nd day after chemotherapy, the rate of constipation and of having first defecation were significantly improved in the intervention group ($p < 0.001$), but the difference of the satisfaction and 'almost no loose stools without purgative use' in Rome III criteria were not significantly changed.<br>On the 5th day, except for 'the sensation of anorectal obstruction' and 'requirement of manual assistance' in Rome III criteria, constipation was significantly improved in the study group ($p < 0.001$); however satisfaction was higher in the control group ($p < 0.001$).<br>At discharge, patients who required laxatives were less in the intervention group ($p < 0.001$)

ASK-12: Adherence Starts with Knowledge; BFI: Brief Fatigue Inventory; BPI: Brief Pain Inventory; BPI-C: Brief Pain Inventory—Chinese version; BQ-SF: Barriers Questionnaire—Short Form; CARE-Q: Caring Assessment Instrument; CASC: Comprehensive Assessment of Satisfaction with Care; CBI-24: Caring Behaviours Inventory—24; CCS: Conviction and Confidence Scale; CES-D: Center for Epidemiological Studies—Depression Scale; CINV: chemotherapy-induced nausea and vomiting; COSTaRS: Pan-Canadian Oncology Symptom Triage and Remote Support; CRI: central venous catheter-related infection; CST: Communication Skills Training; CVC: central venous catheter; DGSS: Dutch General Self-efficacy Scale; EORTC QLQ-C30: European Organisation for Research and Treatment of Cancer Quality of Life Questionnaire—Cancer 30; EORTC QLQ-C15-PAL: European Organisation for Research and Treatment of Cancer Quality of Life Questionnaire—Core 15—Palliative; ESDS: Enforced Social Dependency Scale; F: female; FACT-F: Functional Assessment of Cancer Therapy Fatigue; FACT-G: Functional Assessment of Cancer Therapy-General; FACIT-Sp: Functional Assessment of Chronic Illness Therapy-Spiritual; GAD-7: Generalized Anxiety Disorder seven-item scale; HADS: Hospital Anxiety and Depression Scale; HCAHPS: Hospital Consumer Assessment of Healthcare Providers and Systems; HCI: Holistic Caring Inventory; HSCT: hematopoietic stem cell transplantation; ICS: Individualized Care Scale; INVR: Rhodes Index of Nausea, Vomiting and Retching; JCI: Joint Commission International; M: male sex; MASCC: Multinational Association of Supportive Care in Cancer; MDASI: MD Anderson Symptom Inventory; MDASI-NH: MD Anderson Symptom Inventory—Head and Neck; MSAS: Memorial Symptom Assessment Scale; MUIS-C: Mishel Uncertainty in Illness Scale—Community Form; N: number; NA: not available; NCCN DT: National Comprehensive Cancer Network's Distress Thermometer; NCSR: National Center for Surveys and Research; NIC: nursing outcome classification; NKASRP: Nurses' Knowledge and Attitudes Survey Regarding Pain; NOC: Nursing Outcome Classification; NSC: Nurse Specific Satisfaction with Care; NSO: Nursing-Sensitive Outcomes; OAG: Oral Assessment Guide; ONMDS: Oncology Nursing Minimum Data Set; p:

p-value; PAM: Patient Activation Measure; PBS: Patient Barriers Survey; PGI: Press Ganey Inpatient survey; PGO: Press Ganey Outpatient survey; PHQ-9: Patient Health Questionnaire; PMI: Pain management intervention; PPQ: Patient Pain Questionnaire; PROM: Patient-Reported Outcome Measures; PASQOC: Patient Satisfaction and Quality in Oncological Care; PSCC: Patient Satisfaction With Cancer Care; PSN-1: the Patient Satisfaction With Interpersonal Relationship With Navigator; PSNCQQ: Patient Satisfaction with Nursing Care Quality Questionnaire; PSQ: Patient Satisfaction Questionnaire; PSS: Patient Satisfaction Scale; QOL: Quality Of Life; QONCS: Quality Oncology Nursing Care Scale; QPP: Quality from the Patient's Perspective; RCT: randomized controlled trial; RPS-POQ: Revised American Pain Society—Patient Outcome Questionnaire; SD: standard deviation; SDS: Symptom Distress Scale; SEMCD 6: Self-Efficacy for Managing Chronic Disease Scale; SES: Symptom Experienced Scale; SF-8: Short Form 8 Health Survey; SF-12: Short Form 12-item Health Survey; SF-36: Medical Outcomes Study 36-item Short Form Survey; SMAT: Symptom Management Analysis Tool; SPSI-R: Social Problem Solving Skills—Revised; SSL: Social Support List; STAI-state: State-Trait Anxiety Scale: state version; T0: outcome collection at baseline; T1/T2/T3/T4: outcome collection at some point of the study period; UK: United Kingdom; USA: United States of America: VAS: Visual Analogue Scale.
* multidisciplinary outcome, referred even to other healthcare professionals (e.g., nurse assistants, physicians, …).

Table S4. Summary of NSOs (N = 151) and characteristics from the available studies (N = 60).

NSOs: Categories (n, %) and Sub-categories (n, %)*	Study design (n of studies)	Studied population: N, mean age, prevailing gender (in all n of studies)<br><br>Geographical area (n of studies)	Primary tumour as solid cancer or haematological malignancy (n of studies)	Setting involved in the study (n of studies)
Satisfaction and perception of nursing care received (32, 21.2%):<br><br>satisfaction (19, 12.6%),* experiences and perceptions of care (11, 7.2%),* quality of the relationship with nurses (2, 1.3%)*	Pre/post-test study (5)<br>Prospective and descriptive study (4)<br>RCT (2)<br>Quasi-experimental study (2)<br>Cross-sectional study (2)<br>Qualitative study (2)<br>Scoping review (1)<br>Secondary analysis of a RCT (1)<br>Pre/post-test mixed-method study (1)<br>Retrospective study (1)<br>Descriptive, cross-sectional, correlational study (1)<br>Descriptive cohort study with historic control (1)<br>Multi-site descriptive study (1)<br>Prospective audit and retrospective survey (1)<br>Exploratory qualitative interview study (1)<br>Interpretative qualitative study (1)<br>Protocol of a multicentre RCT (1)<br>Protocol of a mixed-method study of a complex intervention (1)	N = 10,253 (26), NA (3)<br>Mean age = 62.0 years (15), NA (14)<br>F = 5,622 (61.3%) (21), NA (8)<br><br>Europe (16)<br>USA (7)<br>Asia (2)<br>Australia (2)<br>Canada (1)<br>Middle East (1)	Solid cancer (10)<br>Solid cancer and haematological malignancy (6)<br>Haematological malignancy (5)<br>NA (8)	Hospital units (13)<br>Outpatient setting (6)<br>Hospital and outpatient setting (4)<br>Home (2)<br>Hospital units and home (1)<br><br>Cancer Registry (1)<br>Leukaemia Foundation (1)<br>NA (1)
Nursing care process quality (18, 11.9%):<br><br>quality of care received (5, 3.3%), timing (5, 3.3%), caring and uncaring behaviours (4, 2.6%),* access to care (2, 1.3%), acceptability and accessibility of cancer service (1, 0.6%),* disposition at discharge (1, 0.6%)	Pre/post-test study (4)<br>Cross-sectional study (2)<br>Descriptive correlational (2)<br>Cross-sectional and descriptive correlational study (1)<br>Prospective study (1)<br>Retrospective cohort study (1)<br>Descriptive cohort study with historic control (1)	N = 3,884<br>Mean age = 60.8 years (9), NA (7)<br>F = 1,819 (57.1%) (13), NA (3)<br><br>Europe (7)<br>USA (6)<br>Australia (2)<br>Asia (1)	Solid cancer (6)<br>Solid cancer and haematological malignancy (4)<br>Haematological malignancy (3)<br>NA (3)	Hospital units (9)<br>Outpatient setting (2)<br>Community-controlled health service (1)<br>Home (1)<br>Hospital units and home (1)<br>Hospital units and outpatient centres (1)<br>NA (1)

	Qualitative study based on grounded theory (1)<br>Exploratory descriptive study (1)<br>Phenomenological qualitative study (1)<br>Narrative review (1)			
Psychological distress (11, 7.3%):<br><br>general (7, 4.6%), depression (2, 1.3%),* anxiety (2, 1.3%)	Protocol of a multicentre RCT (3)<br>Scoping review (2)<br>Prospective study (2)<br>Pre/post-test design study (1)<br>Cross-sectional prospective pilot study (1)<br>Correlational descriptive study (1)	N = 1,223 (8), NA (2)<br>Mean age = 53.5 years (6), NA (4)<br>F = 553 (59.0%) (7), NA (3)<br><br>Europe (5)<br>USA (3)<br>Asia (1)<br>Australia (1)	Solid tumour (3)<br>Solid tumour and haematological malignancy (3)<br>Haematological malignancy (2)<br>NA (2)	Hospital units (7)<br>NA (2)<br>Outpatient setting (1)
Experiences with therapy-related side effects (11, 7.3%):<br><br>general symptoms (8, 5.3%),* symptom distress (3, 2.0%)	RCT (5)<br>Scoping review (1)<br>Pre/post-test study (1)<br>Quasi-experimental study (1)<br>Protocol of a mixed-method study of a complex intervention (1)<br>Longitudinal, prospective study with quantitative–qualitative approach (1)	N = 1,207 (8), NA (2)<br>Mean age = 60.6 years (5), NA (5)<br>F = 623 (63.6%) (5), NA (5)<br><br>USA (5)<br>Europe (2)<br>Asia (1)<br>Canada (1)<br>South America (1)	Solid cancer (5)<br>NA (3)<br>Solid cancer and haematological malignancy (2)	Hospital units (3)<br>Outpatient setting (5)<br>Home (1)<br>NA (1)
Health care system utilization (10, 6.6%):<br><br>emergency department visits (4, 2.6%), hospital admissions/hospitalizations (2, 1.3%), referrals (1, 0.6%), clinical appointments/visits (3, 2.0%)	RCT (2)<br>Pre/post-test study (1)<br>Secondary analysis of a pre-post study (1)<br>Prospective pre/post-test study (1)<br>Quality improvement project (1)	N = 1,730<br>Mean age = 62.9 years (5), NA (1)<br>F = 457 (63.9%) (3), NA (3)<br><br>USA (4)<br>Canada (2)	Solid cancer (6)	Hospital units (1)<br>Outpatient setting (3)<br>Home (2)
Pain (9, 6.0%):<br><br>resolution/reduction (7, 4.6%),* satisfaction with pain management (2, 1.3%)	Cross-sectional and descriptive correlational study (3)<br>RCT (2)<br>Scoping review (1)<br>Pre/post trial of a quality project (1)<br>Prospective and descriptive study (1)	N = 1,117 (7), NA (1)<br>Mean age = 53.9 years (4), NA (3)<br>F = 360 (52.2%) (5), NA (3)<br><br>Europe (3)<br>USA (2)<br>Asia (1)<br>Australia (1)<br>Middle East (1)	Solid cancer and haematological malignancy (3)<br>Solid cancer (2)<br>NA (2)	Hospital units (3)<br>Hospital and outpatient setting (2)<br>Outpatients setting (1)<br>NA (1)

Health-related quality of life (8, 5.2%)	RCT (3)<br>Scoping review (1)<br>Pre/post-test mixed-method study (1)<br>Secondary analysis of a prospective study (1)<br>Cross-sectional, retrospective program evaluation using a mixed-methods approach (1)<br>Protocol of a multicentre RCT (1)	N = 989 (7), NA (1)<br>Mean age = 58.9 years (5), NA (3)<br>F = 424 (60.4%) (6), NA (2)<br><br>Europe (3)<br>Asia (2)<br>USA (2)<br>Canada (1)	Solid tumour (3)<br>NA (2)<br>Haematological malignancy (1)<br>Solid tumour and haematological malignancy (1)	Outpatient setting (3)<br>Hospital units (2)<br>Home (1)<br>NA (1)
Barriers and facilitators to intervention adherence/symptoms (5, 3.3%)*	RCT (2)<br>Descriptive, cross-sectional, correlational study (1)<br>Cross-sectional, retrospective program evaluation using a mixed-methods approach (1)<br>Mixed methods (1)	N = 564<br>Mean age = 58.3 years (3), NA (2)<br>F = 210 (59.8%) (3), NA (2)<br><br>USA (3)<br>Australia (1)<br>Middle East (1)	Solid cancer (3)<br>Solid cancer and haematological malignancy (2)	Hospital units (2)<br>Outpatient setting (2)<br>Hospital and outpatient setting (1)
Role functioning (4, 2.6%):<br><br>functional status (3, 2.0%),* ability to function independently (1, 0.6%)	RCT (2)<br>Protocol of a multicentre RCT (1)<br>Pre/post-test study (1)	N = 789 (4)<br>Mean age = 59.1 years (2), NA (2)<br>F = 358 (56.5%) (3), NA (1)<br><br>Europe (1)<br>USA (3)	Solid cancer (3)<br>Haematological malignancy (1)	Hospital units (4)
Knowledge in (4, 2.6%):<br><br>understanding diagnosis and disease management (2, 1.3%), managing chronic cancer pain (1, 0.6%), medications and side effects (1, 0.6%)	Pre/post-test design study (2)<br>RCT (1)<br>Prospective and descriptive study (1)	N = 592<br>Mean age = 56.4 years (2) (2 NA)<br>F = 226 (56.0%) (3), NA (1)<br><br>USA (3)<br>Australia (1)	Solid cancer (2)<br>NA (1)<br>Solid tumour and haematological malignancy (1)	Hospital units (3)<br>Hospital and ambulatory units (1)
Self-efficacy (4, 2.6%)	RCT (2)<br>Pre/post-test mixed-method study (1)<br>Protocol of a mixed-method study of a complex intervention (1)	N = 371 (3), NA (1)<br>Mean age = 60 years (1), NA (3)<br>M = 100 (54.9%) (2), NA (2)<br><br>Europe (2)<br>Australia (1)<br>USA (1)	NA (2)<br>Solid cancer (2)	Home (2)<br>Hospital units (1)<br>Outpatient setting (1)
Fatigue (3, 2.0%)	Scoping review (1)<br>RCT (1)<br>Pre/post-test study (1)	N = 230 (2), NA (1)<br>Mean age = 60.0 years (2), NA (1)<br>F = 150 (65.2%) (2), NA (1)	Solid cancer (2)<br>NA (1)	Hospital units (1)<br>NA (1)<br>Outpatient setting (1)

		Asia (1)<br>Canada (1)<br>Europe (1)		
Health status (3, 2.0%)*	RCT (2)<br>Cross-sectional, retrospective program evaluation using a mixed-methods approach (1)	N = 437<br>Mean age = 61.2 years (2), NA (1)<br>F = 137 (55.2%) (2), NA (1)<br>USA (2)<br>Australia (1)	Solid cancer (3)	Outpatient setting (2)<br>Hospital units (1)
Activation (2, 1.3%)	Cross-sectional study (1)<br>Pre/post-test mixed-method study (1)	N = 1,908 (2)<br>NA (2)<br>F = 1,086 (56.9%) (2)<br>Europe (2)	Solid cancer and haematological malignancy (1)<br>NA (1)	Outpatient setting (1)<br>Home (1)
Awareness of the importance of received intervention (2, 1.3%)	Pre/post-test mixed-method study (1)<br>Prospective study (1)	N = 81<br>Mean age = 71 years (1), NA (1)<br>F = 46 (56.8%)<br>Europe (2)	Haematological malignancy (1)<br>NA (1)	Home (1)<br>Hospital units and home (1)
Clinical effectiveness of intervention delivered (2, 1.3%):<br>capecitabine management (1, 0.6%), incidence of CRIs (1, 0.6%)	RCT (1)<br>Prospective audit and retrospective survey (1)	N = 134<br>Mean age = 68.0 years (1), NA (1)<br>M = 82 (61.2%) (2)<br>Europe (2)	Haematological malignancy (1)<br>Solid cancer (1)	Hospital units (1)<br>Outpatient setting (1)
Nausea and vomiting (2, 1.3%)	Prospective and descriptive study (1)<br>Scoping review (1)	N = 270 (1), NA (1)<br>Mean age = 56 years (1), NA (1)<br>M = 54.8% (1), NA (1)<br>USA (2)	NA (2)	Outpatient setting (1)<br>NA (1)
Oral care (2, 1.3%):<br>situation (1, 0.6%)*, mucositis (1, 0.6%)	Pilot assessment study	N = 16 (1), NA (1)<br>Mean age = 60.7 years (1), NA (1)<br>M = 13 (81.3%) (1), NA (1)<br>Europe (2)	Haematological malignancy (1)<br>NA (1)	Hospital units (1)<br>NA (1)
Comfort (1, 0.6%)	Longitudinal, prospective study with quantitative–qualitative approach	N = 21<br>NA<br>NA	NA	Ambulatory units

		South America		
Concerns (1, 0.6%)	Prospective study	N = 70<br>Mean age = 52 years<br>F = 100.0%<br><br>Europe	Solid tumour	Hospital units
Constipation and satisfaction with bowel movements (1, 0.6%)	RCT	N = 120<br>Mean age = 45.8 years<br>M = 66 (55.0%)<br><br>Asia	Haematological malignancy	Hospital units
Coping (1, 0.6%)	Prospective study	N = 134<br>Mean age = 51.8 years<br>F = 83 (61.9%)<br><br>Europe	Solid cancer and haematological malignancy	Hospital units
Cost (1, 0.6%)	Pre/post-test design study	N = 30<br>Mean age = 64 years<br>F = 25 (83.3%)<br><br>USA	Solid cancer and haematological malignancy	Outpatient setting
Diarrhoea (1, 0.6%)	Scoping review	NA<br>NA<br>NA<br><br>Europe	NA	NA
Fall prevention (1, 0.6%)	Secondary analysis, descriptive retrospective study	N = 2,237<br>Mean age = 55 years<br>F = 1,410 (63.0%)<br><br>USA	NA	Hospital units
Nutrition (1, 0.6%)	Scoping review	NA<br>NA<br>NA<br><br>Europe	NA	NA

Patients' assessment and care (1, 0.6%)	Pre/post-test study	N = 50<br>NA<br>NA<br><br>Europe	Solid cancer and haematological malignancy	Hospital units
Perception of health-related information (1, 0.6%)	Cross-sectional study	N = 1,872<br>NA<br>NA<br><br>Europe	Solid tumour and haematological malignancy	Hospital units
Physiologic complications (1, 0.6%)	Pre/post-test study	N = 874<br>Mean age = 70 years<br>NA<br><br>USA	Solid cancer	Home
Problem solving ability (1, 0.6%)	Protocol of a multicentre RCT	N = 286<br>NA<br>NA<br><br>Europe	Haematological malignancy	Hospital units
Safe medication administration (1, 0.6%)	Scoping review	NA<br>NA<br>NA<br><br>Europe	NA	NA
Sleep disturbance (1, 0.6%)	Scoping review	NA<br>NA<br>NA<br><br>Europe	NA	NA
Social support (1, 0.6%)	Protocol of a multicentre RCT	N = 286<br>NA<br>NA<br><br>Europe	Haematological malignancy	Hospital units
Survival (1, 0.6%)	RCT	N = 375<br>≥ 65 years = 246 (65.6%)<br>F = 195 (52.0%)	Solid cancer	Hospital units

		USA		
Tissue integrity (1, 0.6%)	Secondary analysis, descriptive retrospective study	N = 2,237<br>Mean age = 55 years<br>F = 1,410 (63.0%)	NA	Hospital units
		USA		
Uncertainty (1, 0.6%)	RCT	N = 146<br>Mean age = 60 years<br>M = 82 (56.2%)	Solid cancer	Hospital units
		USA		
Unplanned therapy interruption (1, 0.6%)	RCT	N = 165<br>Mean age = 69.9 years<br>F = 89 (53.9%)	Solid tumour and haematological malignancy	Outpatient setting
		Europe		
Use of sources of information (1, 0.6%)	Prospective study	N = 70<br>Mean age = 52 years<br>F = 70 (100.0%)	Solid tumour	Hospital units
		Europe		

APS POQ: American Pain Society Patient Outcomes Questionnaire; ASK-12: Adherence Starts with Knowledge; BFI: Brief Fatigue Inventory; BPI: Brief Pain Inventory; BPI-C: Brief Pain Inventory—Chinese version; BQ-SF: Barriers Questionnaire—Short Form; CARE-Q: Caring Assessment Instrument; CASC: Comprehensive Assessment of Satisfaction with Care; CBI-24: Caring Behaviours Inventory—24; CES-D: Center for Epidemiological Studies-Depression Scale; CINV: chemotherapy-induced nausea and vomiting; CRI: central venous catheter-related infection; DGSS: Dutch General Self-efficacy Scale; EORTC QLQ-C30: European Organisation for Research and Treatment of Cancer Quality of Life Questionnaire—Cancer 30; EORTC QLQ-C15-PAL: European Organisation for Research and Treatment of Cancer Quality of Life Questionnaire—Core 15—Palliative; ESDS: Enforced Social Dependency Scale; F: female; FACT-F: Functional Assessment of Cancer Therapy Fatigue; FACT-G: Functional Assessment of Cancer Therapy-General; FACT-NH: Functional Assessment of Cancer Therapy-Head and Neck; FACT-NHSI: Functional Assessment of Cancer Therapy-Head and Neck Cancer Symptoms Index; FACIT-Sp: Functional Assessment of Chronic Illness Therapy-Spiritual; GAD-7: Generalized Anxiety Disorder seven-item scale; HADS: Hospital Anxiety and Depression Scale; HCAHPS: Hospital Consumer Assessment of Healthcare Providers and Systems; HCI: Holistic Caring Inventory; ICS: Individualized Care Scale; INVR: Rhodes Index of Nausea, Vomiting and Retching; JCI: Joint Commission International; M: male sex; MDASI: MD Anderson Symptom Inventory; MDASI-NH: MD Anderson Symptom Inventory—Head and Neck; MSAS: Memorial Symptom Assessment Scale; MUIS-C: Mishel Uncertainty in Illness Scale—Community Form; n: number of related studies; N: number; NA: not available; NCCN DT: National Comprehensive Cancer Network's Distress Thermometer; NCSR: National Center for Surveys and Research; NOC: Nursing Outcome Classification; NRS: Numeric Rating Scale; NSC: Nurse Specific Satisfaction with Care; NSO: Nursing-sensitive outcomes; OAG: Oral Assessment Guide; PAM: Patient Activation Measure; PBS: Patient Barriers Survey; PGI: Press Ganey Inpatient survey; PGO: Press Ganey Outpatient survey; PHQ-9: Patient Health Questionnaire; POMS: Profile of Mood States; PPQ: Patient Pain Questionnaire; PASQOC: Patient Satisfaction and Quality in Oncological Care; PSCC: Patient Satisfaction With Cancer Care; PSN-1: the Patient Satisfaction With Interpersonal Relationship With Navigator; PSNCQQ: Patient Satisfaction with Nursing Care Quality Questionnaire; PSQ: Patient Satisfaction Questionnaire; PSS: Patient Satisfaction Scale; QONCS: Quality Oncology Nursing Care Scale; QPP: Quality from the Patient's Perspective; RCT: randomized controlled trial; RPS-POQ: Revised American Pain Society—Patient Outcome Questionnaire; SDS: Symptom Distress Scale; SEMCD 6: Self-Efficacy for Managing Chronic Disease Scale; SES: Symptom Experienced Scale; SF-8: Short Form 8 Health Survey; SF-12: Short Form 12-item Health Survey; SF-36: Medical

Outcomes Study 36-item Short Form Survey; SMAT: Symptom Management Analysis Tool; SPERC: Stanford Patient Education Research Center; SPSI-R: Social Problem Solving Skills—Revised; SSL: Social Support List; STAI-state: State-Trait Anxiety Scale: state version; USA: United States of America; VAS: Visual Analogue Scale.

*For multidisciplinary outcomes, referred also to other healthcare professionals (e.g., nurse assistants, physicians).
